# Inclusion complexes of the steroid hormones 17β-estradiol and progesterone with β- and γ-cyclodextrin hosts: syntheses, X-ray structures, thermal analyses and API solubility enhancements

**DOI:** 10.3762/bjoc.18.184

**Published:** 2022-12-22

**Authors:** Alexios I Vicatos, Zakiena Hoossen, Mino R Caira

**Affiliations:** 1 Centre for Supramolecular Chemistry Research (CSCR), Department of Chemistry, University of Cape Town, Rondebosch 7701, South Africa

**Keywords:** cyclodextrin, complexation, 17β-estradiol, progesterone, solubility, X-ray diffraction

## Abstract

Overcoming the challenges of poor aqueous solubility of active pharmaceutical ingredients (APIs) is necessary to render them bioavailable. This study addresses the poor solubility of two potent steroid hormones, 17β-estradiol (BES) and progesterone (PRO), via their complexation with two water-soluble native cyclodextrins (CDs) namely β-CD and γ-CD. The hydrated inclusion complexes β-CD·BES, β-CD·PRO, γ-CD·BES and γ-CD·PRO were prepared via kneading and co-precipitation, and ^1^H NMR spectroscopic analysis of solutions of their pure complex crystals yielded the host–guest stoichiometries 2:1, 2:1, 1:1 and 3:2, respectively. Both powder X-ray diffraction (PXRD) and single-crystal X-ray diffraction (SCXRD) were employed for focused studies of the isostructurality of the CD complexes with known complexes and structural elucidation of the new complexes, respectively. SCXRD analyses of β-CD·BES, β-CD·PRO and γ-CD·PRO at 100(2) K yielded the first crystal structures of CD complexes containing the hormones BES and PRO, while the complex γ-CD·BES was readily shown to be isostructural with γ-CD·PRO by PXRD. Severe disorder of the encapsulated steroid molecules in the respective channels of the CD molecular assemblies was evident, however, preventing their modelling, but combination of the host–guest stoichiometries and water contents of the four hydrated inclusion complexes enabled accurate assignment of the chemical formulae of these ternary systems. Predicted electron counts for the complexed molecules BES and PRO correlated reasonably well with the complex compositions indicated by ^1^H NMR spectroscopy. Subsequent measurements of the aqueous solubilities of the four complexes confirmed significant solubility improvements effected by encapsulation of the steroids within the CDs, yielding solubility enhancement factors for BES and PRO in the approximate range 5–20.

## Introduction

**Figure 1:**
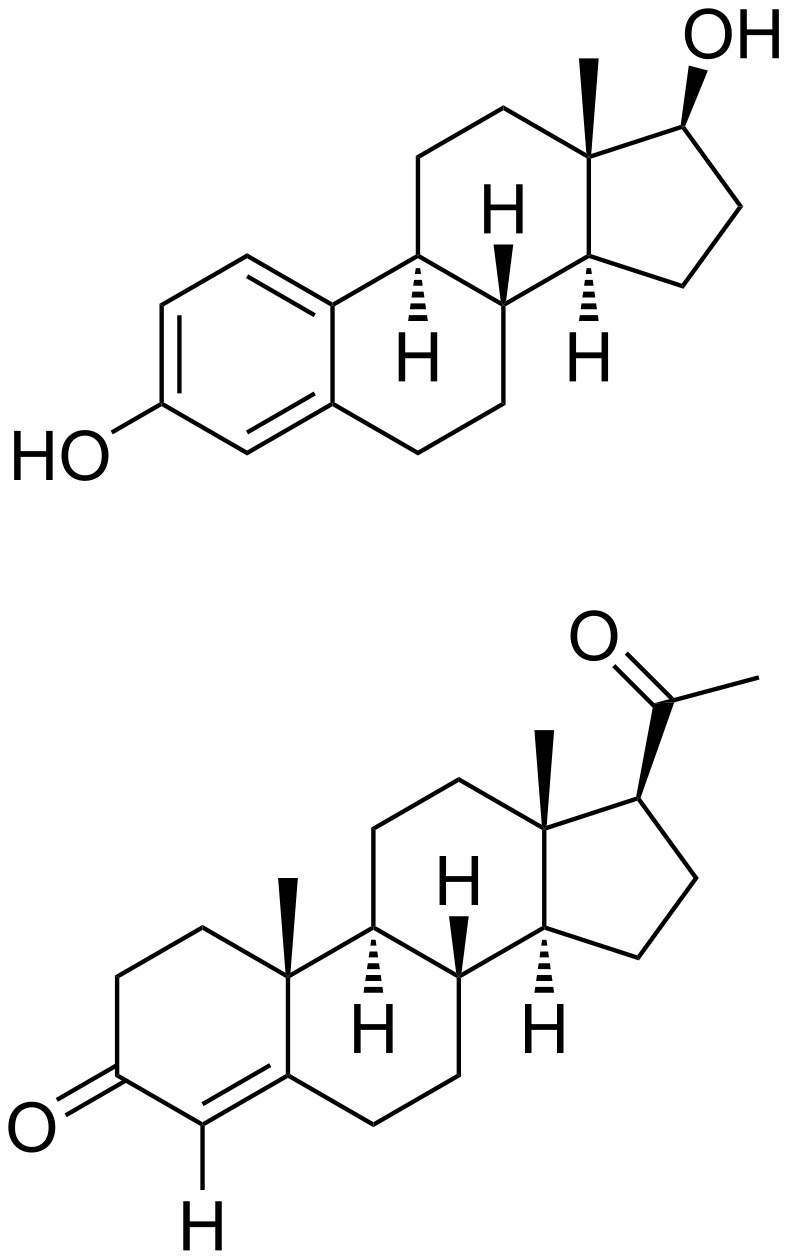
Chemical structures of 17β-estradiol (top) and progesterone (bottom).

The insolubility of active pharmaceutical ingredients (APIs) and other bioactive compounds in aqueous media and the associated challenges for effective drug delivery are well known to have beleaguered the pharmaceutical industry for many decades. However, the complexation of APIs with cyclodextrins (cyclic oligosaccharides) resulting in the formation of inclusion complexes, has proven to be a versatile technology for overcoming not only the poor aqueous solubility of APIs, but also unfavourable properties such as their chemical instability, adverse side effects such as gastrointestinal irritation, and unpleasant tastes and odours [1–5]. Here we report an investigation of the cyclodextrin (CD) inclusion of two notable bioactive compounds (Figure 1), namely the most potent human estrogen, 17β-estradiol (BES) and progesterone (PRO), the steroid hormone associated with female fertility and pregnancy. Both compounds have very low aqueous solubility values (3.6 × 10^−3^ mg·cm^−3^ at 27 °C, and 8.81 × 10^−3^ mg·cm^−3^ at 25 °C, respectively) [6–8]. This drawback hinders their use as medications in hormone replacement therapy and other treatments, and one aim of this study is to enhance their solubilities via their inclusion in selected native (natural) CDs. A commercially available synthetic derivative of BES, ethinylestradiol, which is used as (inter alia) a contraceptive, has an aqueous solubility of 11.3 × 10^−3^ mg·cm^−3^ at 27 °C [6,9], and a significantly higher oral bioavailability relative to that of BES. The steroid hormone PRO is commercially available either in its natural form or as micronized natural progesterone [10].

A substantial volume of research on the topic of CD inclusion of BES and PRO has already been published, the majority of the publications being focused on medicinal applications, for example, using formulations of these drugs with CDs for ophthalmic and nasal delivery [11–21]. The scientific literature abounds with reports featuring the use of a wide variety of analytical and confirmatory techniques [22–28] as well as solubility analyses [29–38] to investigate the physicochemical properties of CD inclusion complexes and multicomponent CD inclusion systems based on numerous derivatised CDs containing BES and PRO, thus confirming the sustained interest in this approach and its significance in drug delivery. Previous studies of the complexation of BES, PRO and related compounds by CDs in the solution phase have typically reported the use of NMR spectroscopy to investigate complex formation, complex structure, stoichiometry and association constants via the detection and measurement of small deviations in the ^1^H NMR chemical shifts for both host and guest molecules upon complexation. PXRD studies were generally used to detect the formation of new crystalline phases following attempts to prepare complexes by spray-drying, freeze-drying, co-grinding, kneading and solution-based crystallization; however, this modest type of ‘fingerprinting’ has not generally been pursued further to deduce important features of CD complexes such as the crystal packing of the CD host molecules. Furthermore, no previous structure determinations via SCXRD analysis of CD complexes of BES and PRO have been reported. Many studies involved phase solubility analyses to determine CD complex formation and association constants. Our previous study of the complexation of the steroidal anticancer agent 2-methoxyestradiol (2ME) by selected CDs [39] yielded two significant positive outcomes, namely a considerable increase in the aqueous solubility and dissolution rate of 2ME derived from its β-CD complex, and for the first time, the determination of the modes of inclusion of a steroidal molecule within the cavities of CD host molecules (dimethylated and permethylated β-CD) by SCXRD. Motivated by these favourable results, we recently prepared the four crystalline inclusion complexes β-CD·BES, β-CD·PRO, γ-CD·BES and γ-CD·PRO with a view to using PXRD, not simply as a routine fingerprint technique, but in a more innovative study of their crystal isostructurality within the series and with published complexes. SCXRD was employed to deduce structural features of the complexes at the molecular level, while the occurrence of severe guest disorder prompted the non-routine application of a method for yielding complex composition from the residual electron density peaks in the respective crystal structures. We carried out analogous studies on the four CD complexes to establish whether they might also display enhanced aqueous solubilities of the included steroids relative to those of the pure drugs. We also present here results of comprehensive thermal characterization of these complexes, with emphasis on the determination of their water contents.

While it is widely known that the solubility of poorly water-soluble steroidal compounds such as BES and PRO may be very significantly enhanced by various derivatised CDs, it was of interest to us to establish to what extent the native host compounds β-CD and γ-CD could improve the aqueous solubilities of BES and PRO, specifically upon dissolution of their solid hydrated inclusion complexes whose accurately derived ternary formulae were established in this study. The relatively low cost of these CDs and in particular the low toxicity of γ-CD are advantageous for potential drug delivery. Furthermore, the amorphous nature of highly water-soluble CD derivatives (e.g., hydroxypropyl-CD and sulfobutyl ether CD) precludes their formation of crystalline inclusion complexes, which is a distinct disadvantage for solid-state structural investigation and complex characterization by X-ray diffraction methods.

## Results and Discussion

### Complex screening

**Figure 2:**
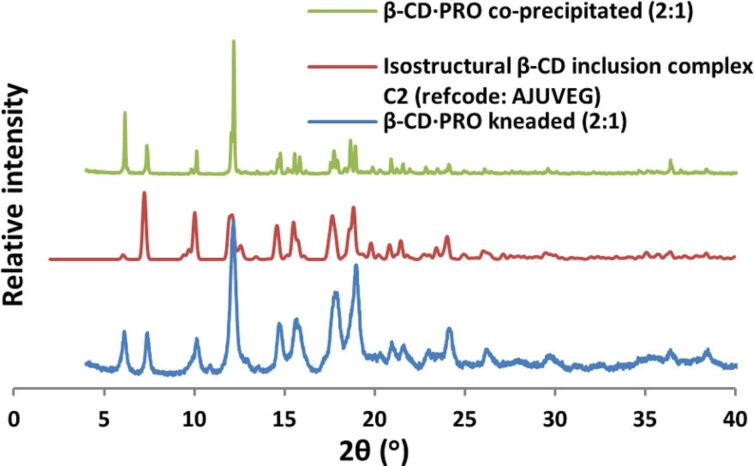
The PXRD patterns of the β-CD·PRO complex produced via kneading (2:1), an isostructural β-CD complex crystallizing in the space group *C*2 (refcode: AJUVEG), and the β-CD·PRO complex produced via co-precipitation (2:1).

**Figure 3:**
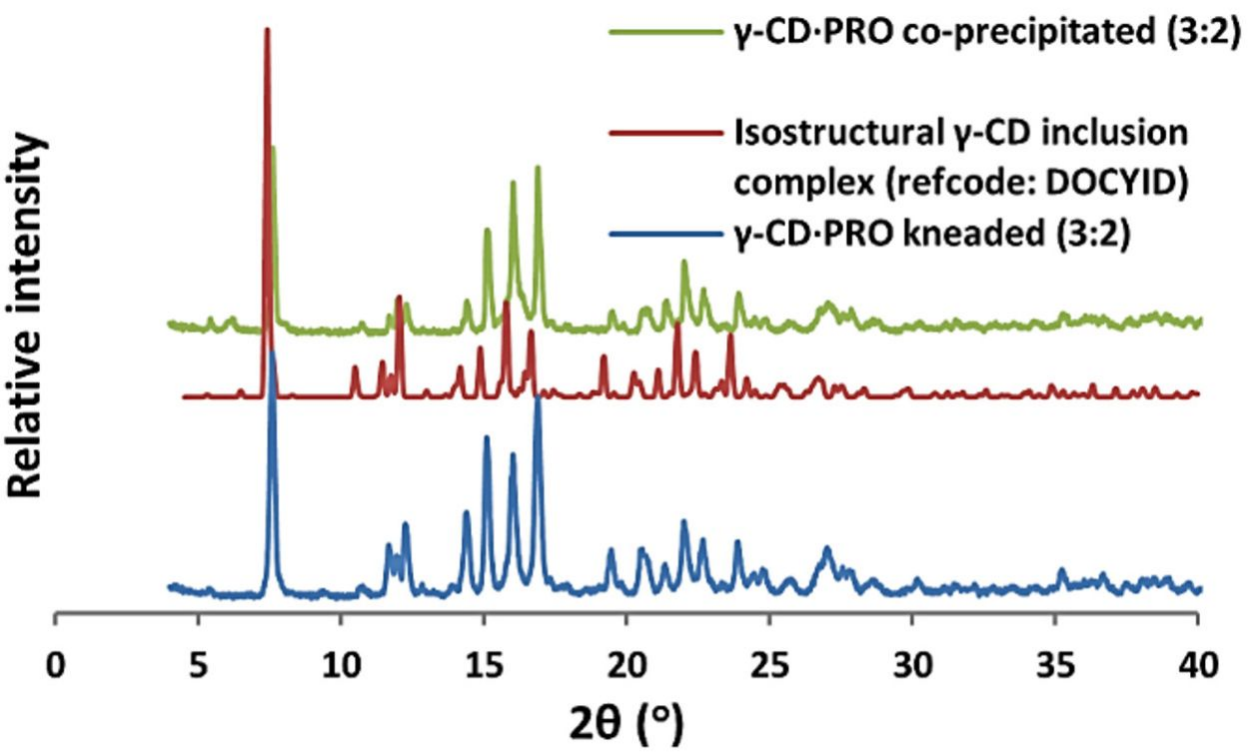
The PXRD patterns of the γ-CD·PRO complex produced via kneading (3:2), an isostructural γ-CD complex crystallizing in the space group *P*42_1_2 (refcode: DOCYID), and the γ-CD·PRO complex produced via co-precipitation (3:2).

The isolation of solid inclusion complexes of BES and PRO with the native cyclodextrins β-CD and γ-CD was successfully achieved by kneading experiments (co-grinding of the respective hosts and guests with water as a medium), the complex identities being subsequently determined unequivocally by powder X-ray diffraction (PXRD). Authentic CD complex formation was deduced from close correlation between the angular peak positions of the products and those of known isostructural CD inclusion complexes retrieved from the Cambridge Structural Database (CSD) [40]. Figure 2 and Figure 3 show representative examples of peak-matching for isostructural complexes [41] and the respective PXRD patterns of β-CD·BES and γ-CD·BES are also available (Figures S1 and S2 in Supporting Information File 1). This method enabled initial assignment of the space group for each new complex and estimation of its unit cell parameters, these data being subsequently confirmed by single crystal X-ray diffraction for those complexes that formed crystals of adequate size and quality.

**Figure 4:**
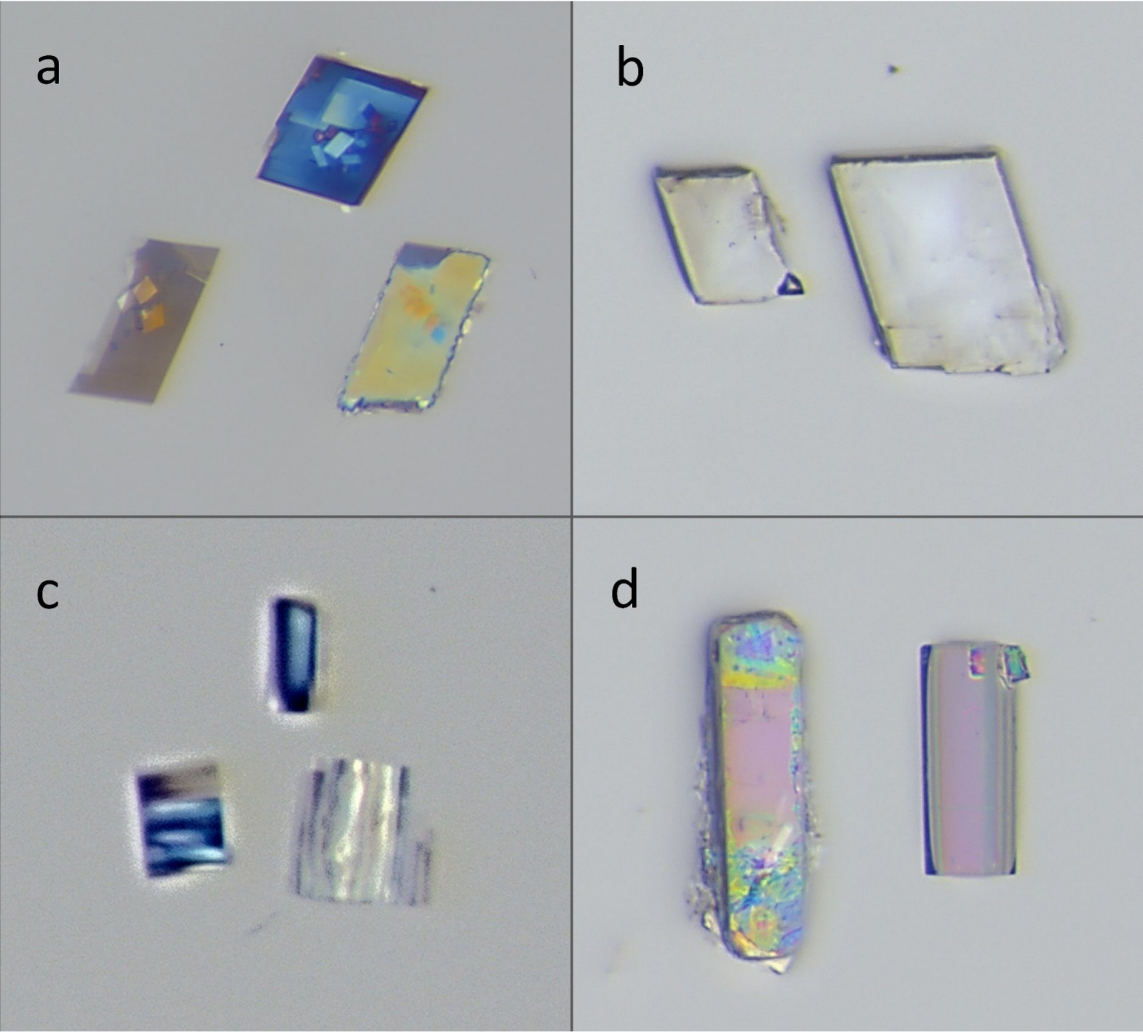
(a) The crystal morphology of β-CD·BES recorded with polarised light. (b) The crystal morphology of β-CD·PRO. (c) The highly magnified crystal morphology of γ-CD·BES. (d) The crystal morphology of γ-CD·PRO.

Co-precipitation experiments yielded single crystals of all four CD inclusion complexes with distinctive transparent monoclinic morphologies for β-CD·BES and β-CD·PRO, and transparent rods with a square cross-section for γ-CD·BES and γ-CD·PRO (Figure 4). PXRD analysis also confirmed that pure crystalline phases were obtained for each complex product via co-precipitation experiments and that each of these was the same crystalline phase as the respective complex product produced via kneading. These single crystals were of adequate quality for SCXRD except for the single crystals of γ-CD·BES, which diffracted very weakly.

### Host–guest stoichiometric determination by ^1^H NMR spectroscopy

The host–guest stoichiometries for these complexes were unambiguously determined via solution-state ^1^H NMR spectroscopy, the samples for the analyses being the complex product materials produced via co-precipitation. The results for the host–guest ratios were as follows: β-CD·BES (2:1), β-CD·PRO (2:1), γ-CD·BES (1:1), γ-CD·PRO (3:2). Fully assigned spectral analyses are also available (Figures S3–S8 and Tables S1–S4 in Supporting Information File 1).

### Thermal analysis

**Figure 5:**
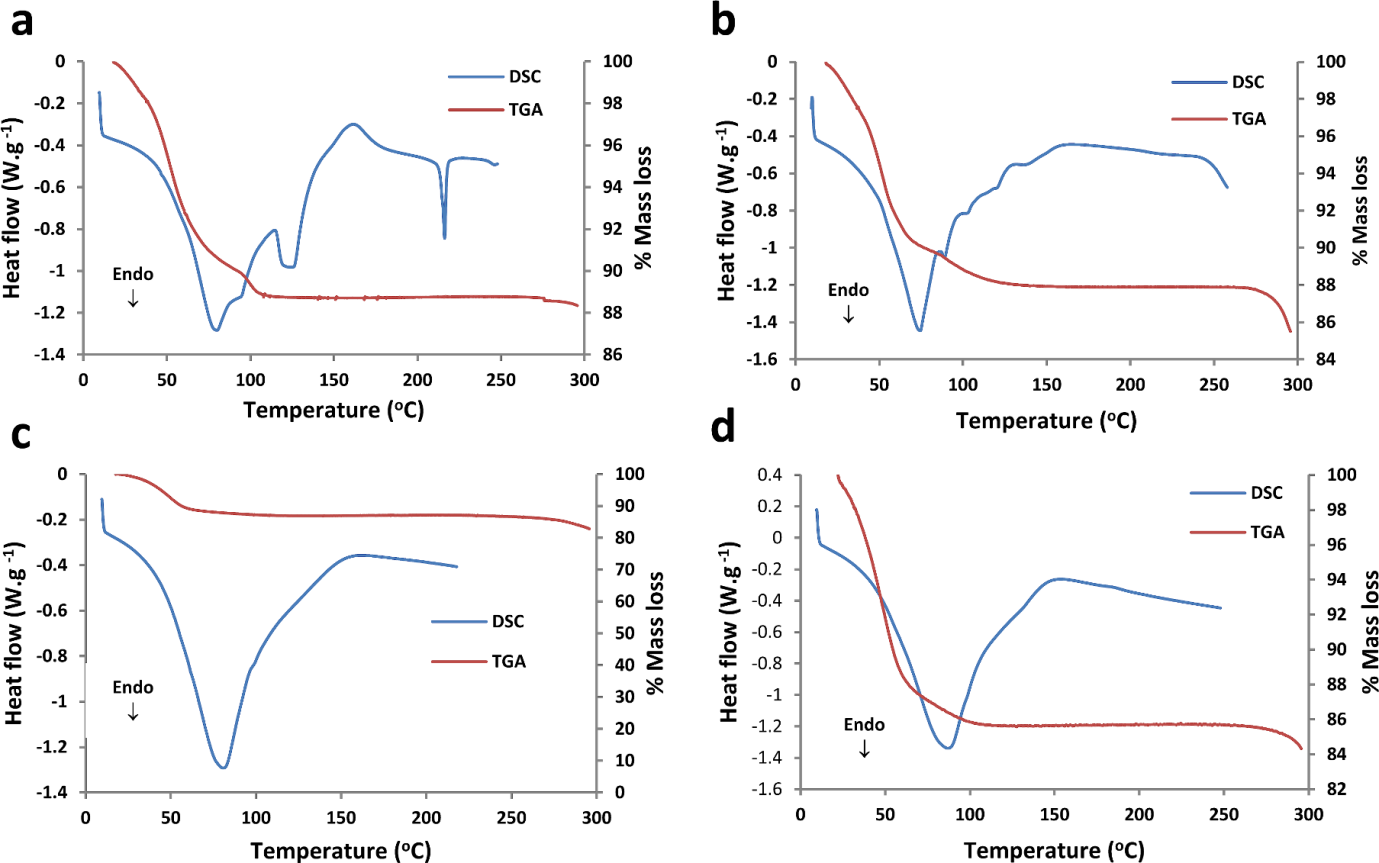
(a) A representative DSC curve (*n* = 2) of β-CD·BES, with the respective TGA curve (*n* = 3); (b) a representative DSC curve (*n* = 2) of β-CD·PRO, with the respective TGA curve (*n* = 2); (c) a representative DSC curve of γ-CD·BES (*n* = 2), with the respective TGA curve as a reference (*n* = 2); (d) a representative DSC curve of γ-CD·PRO (*n* = 3), with the respective TGA curve as a reference (*n* = 3).

The highly reproducible thermogravimetric analyses (TGA) and differential scanning calorimetry (DSC) analyses for the four CD inclusion complexes (Figure 5a–d) primarily indicated their water content and thermal stability, the data for their thermal events being summarised in Table 1. These results were further accompanied by hot stage microscopy (HSM) analyses for all four complexes (Figures S9–S12 in Supporting Information File 1) and variable temperature powder X-ray diffraction (VTPXRD) analyses for β-CD·BES and β-CD·PRO (Figures S13 and S14 in Supporting Information File 1). The precise temperature onset values did not always correlate when comparing the results obtained from the TGA and DSC instruments, but the two methods did involve different sample configurations (samples in open pans vs crimped, vented pans).

**Table 1:** Data for the TGA and DSC thermal events for β-CD·BES, β-CD·PRO, γ-CD·BES, γ-CD·PRO.

CD inclusion complex	TGA	DSC

β-CD·BES	dehydration: temperature range of event: 18.7 ± 1.1 and 120.4 ± 1.3 °C associated mass loss: 11.1 ± 0.2%, corresponding to 8.8 ± 0.2 water molecules per host molecule. decomposition onset: 266.4 ± 5.5 °C	onset temp. (dehydration): 53.2 ± 1.0 °C peak temp. (dehydration): 83.0 ± 4.7 °C onset of shoulder endotherm (dehydration): 91.8 ± 5.1 °C peak shoulder temp. (dehydration): 99.9 ± 5.3 °C onset temp. (dehydration): 119.0 ± 3.4 °C peak temp. (dehydration): 128.9 ± 3.5 °C onset temp. (exotherm): 154.7 ± 2.5 °C peak temp. (exotherm): 158.7 ± 2.0 °C onset temp. (decomp.): 213.4 ± 0.1 °C peak temp. (decomp.): 216.4 ± 0.1 °C

β-CD·PRO	dehydration: temperature range of event: 20.2 ± 2.1 °C and 148.4 ± 0.9 °C associated mass loss: 12.3 ± 0.2% corresponding to 10.0 ± 0.2 water molecules per host molecule. decomposition onset: 267.8 ± 3.1 °C	onset temp. (dehydration): 46.5 ± 0.4 °C peak temp. (dehydration): 74.1 ± 0.2 °C shoulder temp. (dehydration): 89.6 ± 0.5 °C shoulder temp. (dehydration): 103.3 ± 0.2 °C shoulder temp. (dehydration): 121.2 ± 0.6 °C shoulder temp. (dehydration): 138.5 ±0.9 °C onset temp. (phase transition exotherm): 157.1 ± 0.1 °C peak temp. (phase transition exotherm): 162.0 ± 0.2 °C

γ-CD·BES	temperature range of dehydration: 17.8 ± 0.3 to 144.9 ± 0.1 °C associated mass loss for dehydration: 12.7 ± 0.4% corresponding to 12.7 ± 0.4 water molecules per host molecule. onset of decreased dehydration rate (shoulder peak in dTGA): 64.5 ± 0.6 °C decomposition onset: 219.5 ± 2.4	onset temp. (dehydration): 46.7 ± 2.5 °C peak temp. (dehydration): 83.5 ± 2.9 °C

γ-CD·PRO	temperature range of dehydration: 20.6 ± 1.8 °C and 119.6 ± 5.0 °C associated mass for dehydration loss: 14.7 ± 0.3% corresponding to 14.4 ± 0.3 water molecules per host molecule. onset of decreased dehydration rate (shoulder in dTGA): 77.5 ± 4.5 °C decomposition onset: 246.8 ± 2.5 °C	onset temp. (dehydration): 49.3 ± 0.5 °C peak temp. (dehydration): 87.9 ± 0.1 °C shoulder temp. (dehydration): 131.1 ± 0.6 °C

For all four complexes dehydration was initially observed, either as a multi- or single-step process, as confirmed by the respective DSC curves (Figures S15–S18 in Supporting Information File 1) and dTGA curves (Figures S19–S22 in Supporting Information File 1). Other thermal events such as minor phase transitions were observed for β-CD·BES and β-CD·PRO as well as premature decomposition, displayed by β-CD·BES in particular (Table 1). Thus, all CD complexes displayed thermal integrity until relatively high temperatures of at least 100 °C, prior to phase transitions and decomposition, indicating acceptable thermal stability for commercial pharmaceutical applications. Further analytical details regarding the TGA, dTGA and DSC results for each sample are available (Section 5, pp. S15 and S16 in Supporting Information File 1).

### Single crystal X-ray structural analysis

The crystal structures of β-CD·BES, β-CD·PRO and γ-CD·PRO were solved by isomorphous replacement using as trial models the rigid host atom frameworks of the complexes with CSD refcodes AJUVEG, NUFTUE and MUXBIT, respectively. (Single crystal intensity data could not be collected for γ-CD·PRO owing to the poor quality of diffraction from selected crystal specimens). Following least-squares refinements of the respective host atoms in β-CD·BES, β-CD·PRO and γ-CD·PRO and location and refinement of water oxygen atoms, difference electron density (Δρ) peaks with very low magnitudes (generally ≤ 1 e·Å^−3^) appeared within the respective host cavities, indicating severe disorder of the included steroidal guest molecules. This is a common situation for β-CD complexes crystallizing in the space groups *C*2 and *P*42_1_2, in which the host molecules stack with successive host cavities in alignment, generating infinite channels occupied by guest molecules. In the case of β-CD inclusion complexes, guest molecules can assume multiple orientations and positions within the channels having twofold rotational symmetry, rendering them impossible to model. Analogously, regarding γ-CD inclusion complexes, in addition to the possibility of guest molecules assuming random orientations and locations within the infinite channels, the latter possess four-fold rotational symmetry and thus, unless the guest molecule also possesses this symmetry, it will be severely disordered and hence not able to be modelled. In many instances, the pursuance of the crystal structure of an inclusion compound that suffers from such severe guest disorder is eventually abandoned owing to the uninterpretable, unassigned electron density that results in unacceptable values for X-ray refinement parameters.

However, to circumvent this problem we used a well-known method that enables integration of multiple unassigned Δρ peaks located in crystal voids, namely the SQUEEZE routine [42] implemented in the program PLATON [43]. Following automatic location of prominent voids in the unit cell of each crystal structure, this procedure provides an estimate of the total number of residual electrons they contain, which in these complexes would correspond to those of the included steroidal molecules. From these respective electron counts, it was thus possible to estimate the host–guest stoichiometric ratios in each complex for comparison with the accurate stoichiometry determinations using ^1^H NMR spectroscopy. In addition, final X-ray refinement parameters improved following application of the SQUEEZE routine. Although this procedure is most commonly used for dealing with disordered solvent molecules for which an atomistic model is not achievable from the observed residual Δρ peaks, we found that its extension to the treatment of significantly larger molecules (viz. disordered steroidal guests BES and PRO) yielded quite reasonable electron count values for their contributions. Details of the estimations of the host–guest stoichiometries for the β-CD·BES, β-CD·PRO and γ-CD·PRO complexes based on these values are provided (Section 6, p. S21 in Supporting Information File 1). The host–guest stoichiometric estimates obtained consequently correlated with the results from the ^1^H NMR analyses to varying degrees from very good to fair.

PXRD patterns generated from the final single crystal X-ray structures were in good agreement with the respective PXRD patterns of the CD complexes formed via co-precipitation (Figure S23 in Supporting Information File 1) indicating that the single crystal specimens selected for X-ray analysis are representative of their corresponding bulk materials. The crystallographic data for these complexes are summarised in Table 2. The hydrated CD inclusion complexes of 17β-estradiol and progesterone are ternary systems and their accurate chemical formulae listed in Table 2 were established by combining ^1^H NMR data (for host–guest ratios) and TGA (for crystal water contents). The same method was employed to determine the chemical formula for γ-CD·BES (namely C_48_H_80_O_40_·C_21_H_30_O_2_·12.7H_2_O) for which no single crystals of adequate diffraction quality were isolated.

**Table 2:** The crystal data and refinement parameters for the β-CD·BES, β-CD·PRO and γ-CD•PRO inclusion complexes.

Parameter	β-CD·BES	β-CD·PRO	γ-CD·PRO

complex formula	2(C_42_H_70_O_35_)·(C_18_H_24_O_2_) ·17.7H_2_O	2(C_42_H_70_O_35_)·(C_21_H_30_O_2_) ·20H_2_O	C_48_H_80_O_40_·(C_21_H_30_O_2_)_0.67_ ·14.4H_2_O
formula weight (g·mol^−1^)	2859.40	2944.72	1765.64
temperature (K)	100(2)	100(2)	100(2)
wavelength (Å)	0.71073	0.71073	0.71073
crystal system	monoclinic	monoclinic	tetragonal
space group	*C*2	*C*2	*P*42_1_2
*a* (Å)	19.087(2)	18.9408(8)	23.6991(9)
*b* (Å)	24.427(2)	24.540(1)	23.6991(9)
*c* (Å)	15.581(1)	15.6585(8)	22.896(1)
α (^o^)	90	90	90
β (^o^)	109.555(2)	109.161(1)	90
γ (^o^)	90	90	90
volume (Å^3^)	6845.4(10)	6875.1(6)	12860(1)
*Z*	2	2	6
calculated density (g·cm^−3^)	1.387	1.422	1.368
μ (mm^−1^)	0.123	0.126	0.121
F (000)	3056	3152	5678
crystal size (mm)	0.48 × 0.44 × 0.10	0.34 × 0.24 × 0.16	0.20 × 0.21 × 0.36
θ-range scanned (^o^)	1.39–26.87	2.74–28.34	1.24–26.41
index range	*h*: −24, 24; *k*: −30, 30; *l*: −19, 19	*h*: −25, 25; *k*: −32, 32; *l*: −20, 20	*h*: −29, 29; *k*: −29, 29; *l*: −27, 28
no. of reflections collected	75496	150313	118399
no. of unique reflections	14663	17104	13196
data completeness (%)	99.5	99.6	99.7
data/restraints/parameters	14663/19/756	17104/28/750	13196/12/365
S (goodness-of-fit on F^2^)	1.038	1.021	1.099
final R indices R_1_, wR_2_, [I > 2σ(I)]	0.0829, 0.2352	0.0782, 0.2174	0.0969, 0.2319
R indices, all data (R_1_, wR_2_)	0.1043, 0.2593	0.0903, 0.2306	0.0996, 0.2337
largest diff. peak and hole (e.Å^−3^)	0.72, −1.30	0.83, −0.43	0.85, −0.46
CCDC deposition numbers	2213533	2213538	2213558

**Figure 6:**
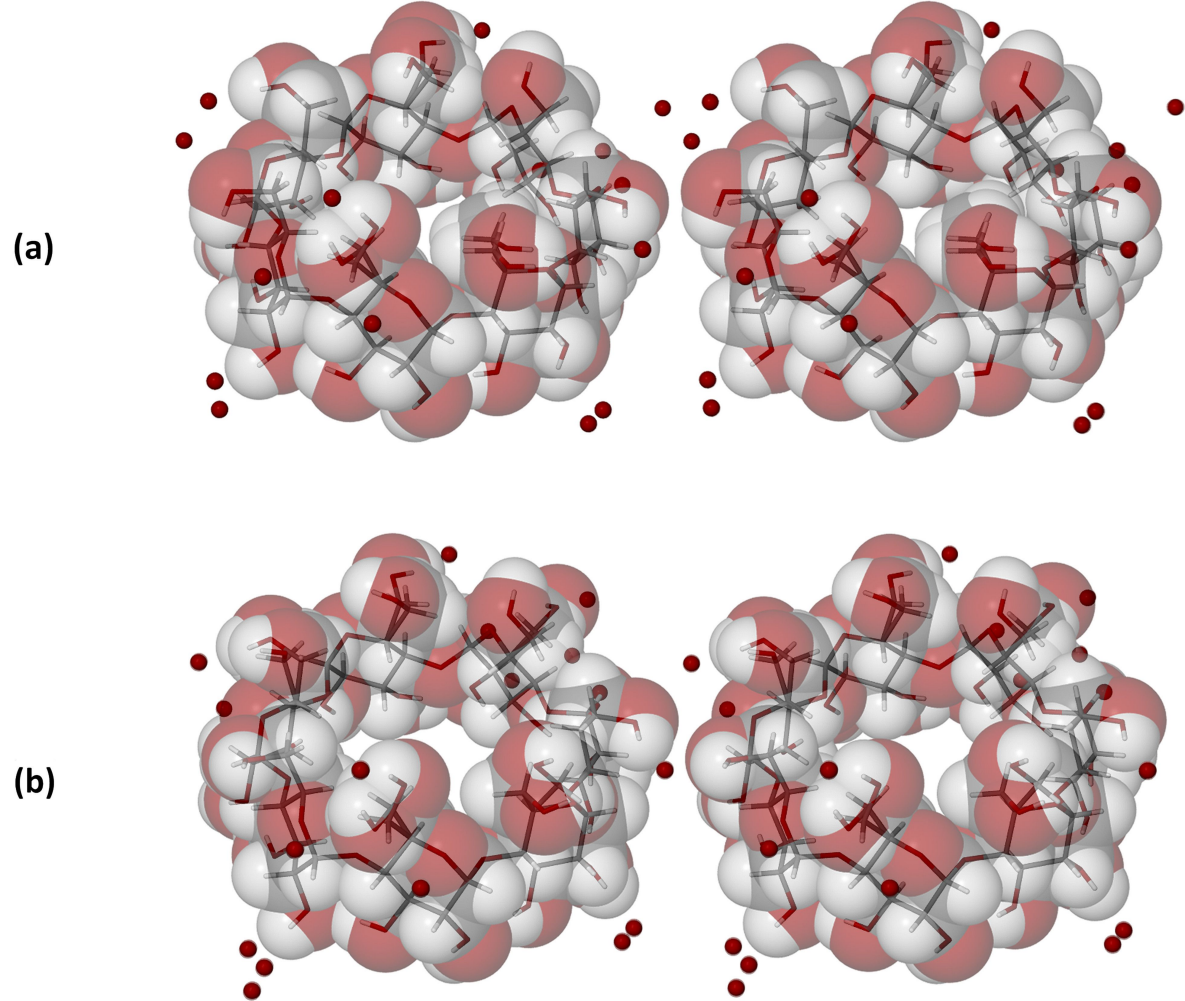
Stereoscopic views of the host molecule and water oxygen atoms in the ASUs of (a) β-CD·BES and (b) β-CD·PRO displaying their ‘empty’ host cavities.

The asymmetric units (ASUs) of β-CD·BES and β-CD·PRO (Figure 6) each consist of a single β-CD molecule, one severely disordered guest molecule (either 17β-estradiol for β-CD·BES, or progesterone for β-CD·PRO), and water molecules distributed over 15 sites for β-CD·BES, and 17 sites for β-CD·PRO. The water contents for β-CD·BES and β-CD·PRO based on their X-ray analyses were 9.1 and 9.9 water molecules per β-CD molecule, which correlated well with the TGA results (Table 1). Minor disorder was observed for the host molecules of both complexes, while in contrast, severe disorder for their respective guest molecules was evident, despite the intensity data being collected at 100(2) K. Lastly, reasonable hydrogen bonding distances were observed between the assigned oxygen atoms of the water molecules and neighbouring water oxygen atoms, or the host molecules.

**Figure 7:**
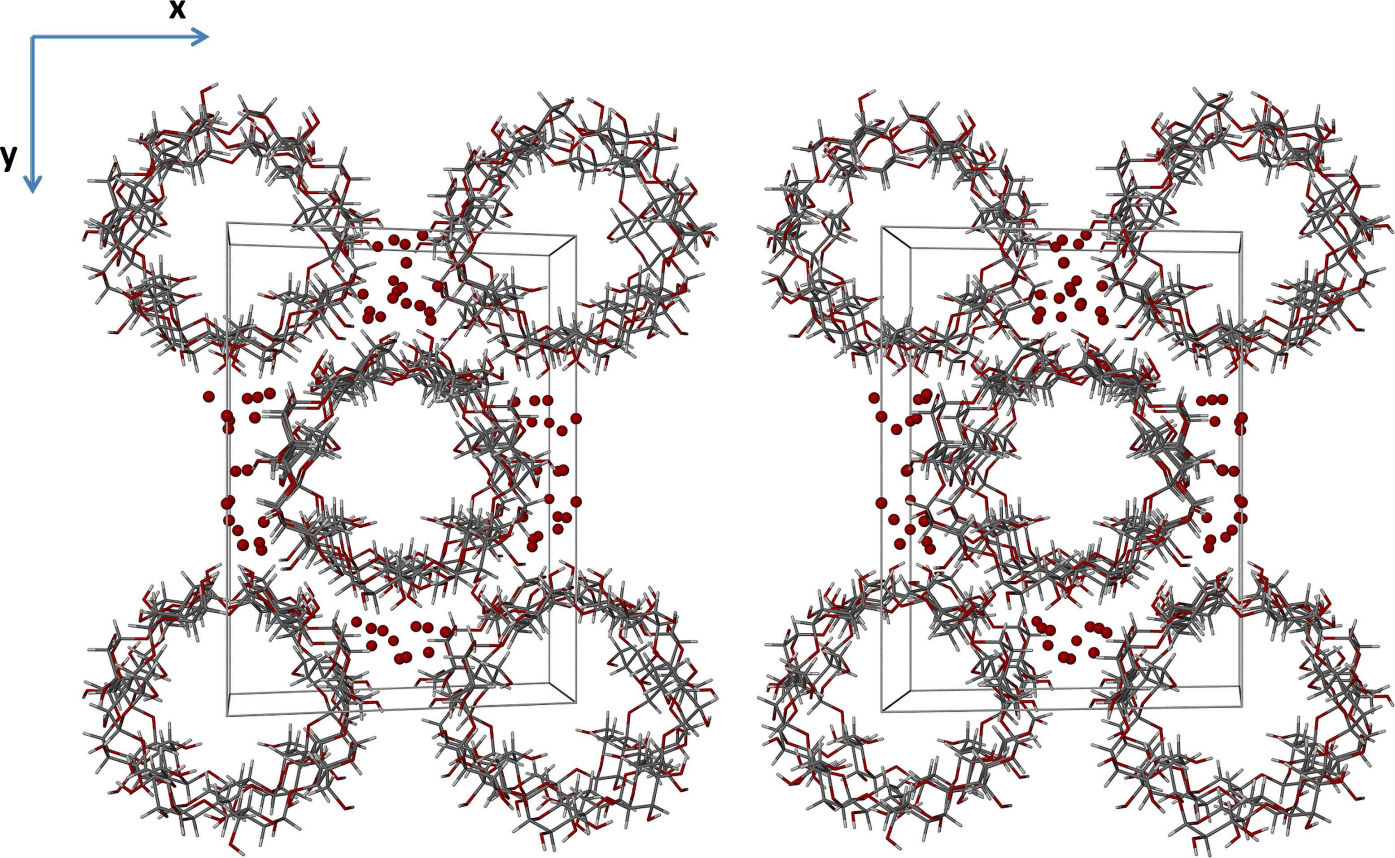
A stereoscopic view down the *c*-axis displaying the packing arrangement for β-CD·BES.

**Figure 8:**
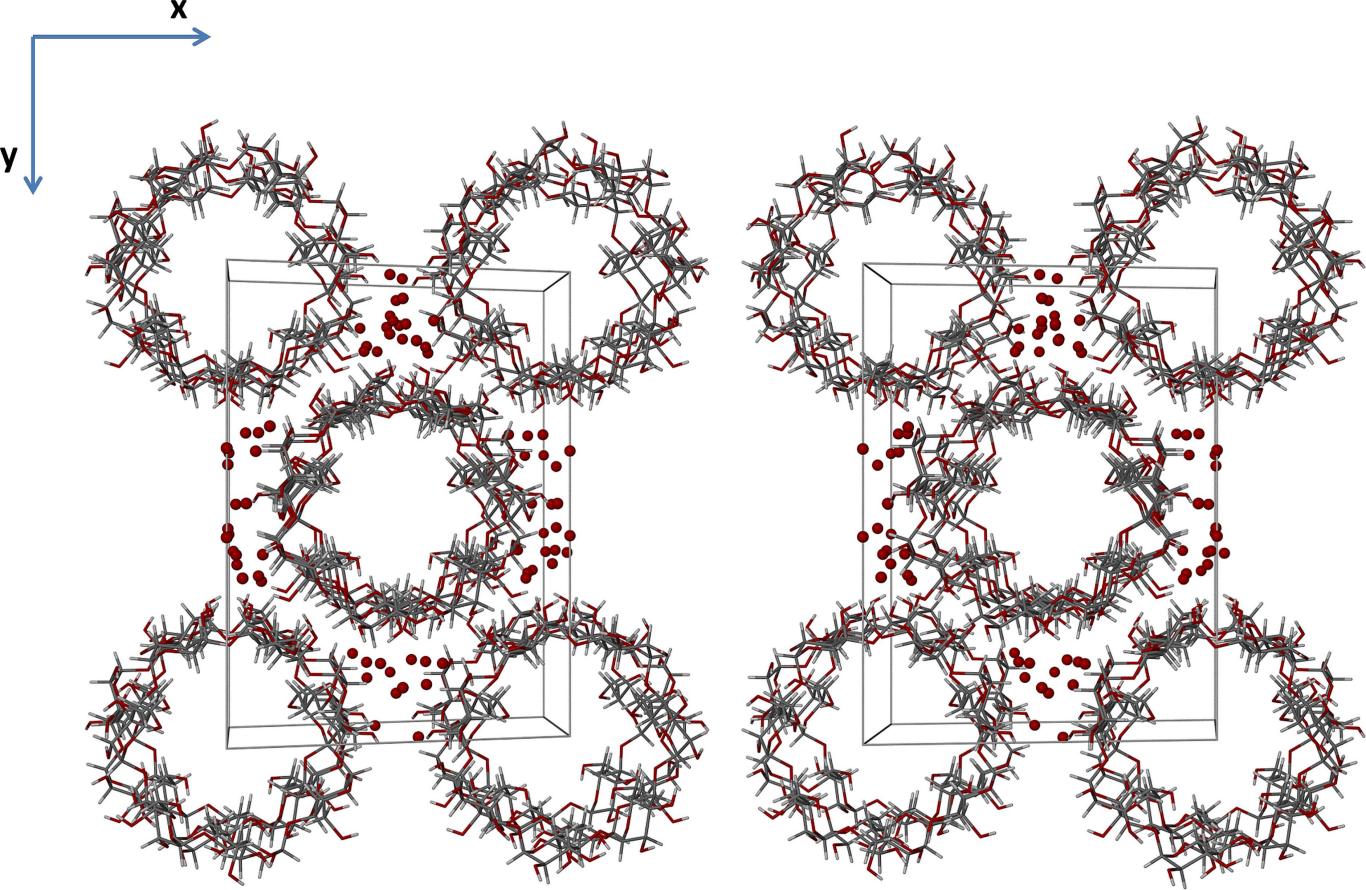
A stereoscopic view down the c-axis displaying the packing arrangement for β-CD·PRO.

Isostructurality of the host molecules in the two β-CD complexes is evident from the figures below, but it is also notable that isostructurality extends beyond these molecules to include numerous water oxygen atoms as well. The stacking arrangements of the dimeric host molecule units of β-CD inclusion complexes crystallizing in the space group *C*2 are well-known [44], these arrangements resulting in endless channel formation occurring in the crystal structures of β-CD·BES and β-CD·PRO (Figure 7 and Figure 8, depicting stereoscopic views observed down the *c*-axis). Furthermore, it is evident that the water molecules which occupy the interstitial spaces between the columns reinforce this assembly of complex units via multiple O–H···O hydrogen bonds. These depictions also indicate that there are four β-CD molecules in the unit cell, corresponding to two dimeric complex units per cell and again reveal the similarity in the conservation of water molecule sites in the two isostructural complexes.

The detailed host conformations are defined by numerous, relevant geometrical parameters that include measures of the angular tilts of the individual glucose rings relative to the seven-fold axes of the β-CD molecules (Figures S24–S25 and Tables S5–S6 in Supporting Information File 1). The narrower ranges of the tilt angles for β-CD·BES and β-CD·PRO relative to the tilt angle range for uncomplexed hydrated β-CD (4.5°–27.0°) [45] indicate that additional distortion took place with host–guest complexation, as a consequence of a mutual-induced fit of host and guest molecules [39].

**Figure 9:**
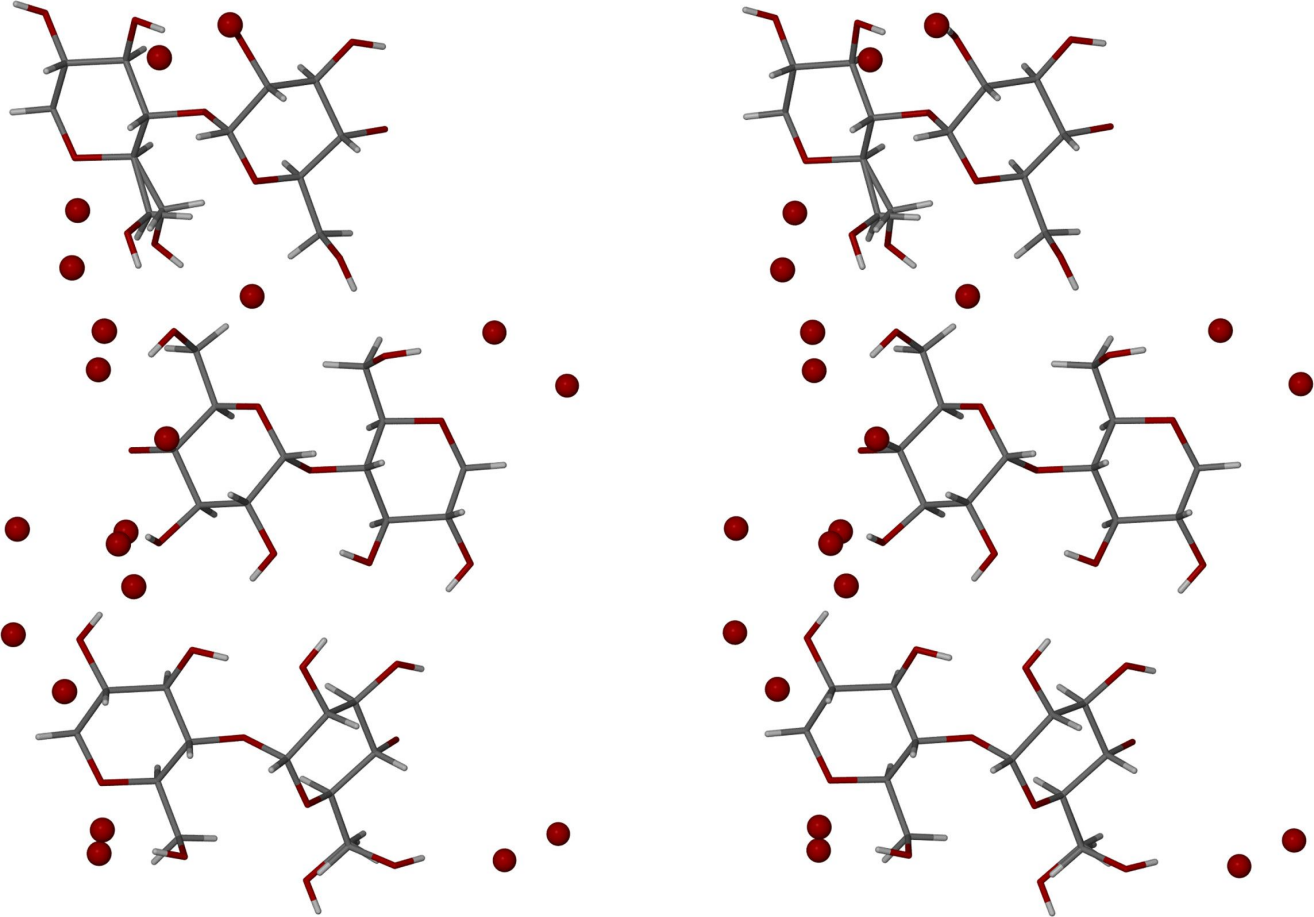
A stereoscopic view of the host atoms and water oxygen atoms in the ASU of γ-CD·PRO.

**Figure 10:**
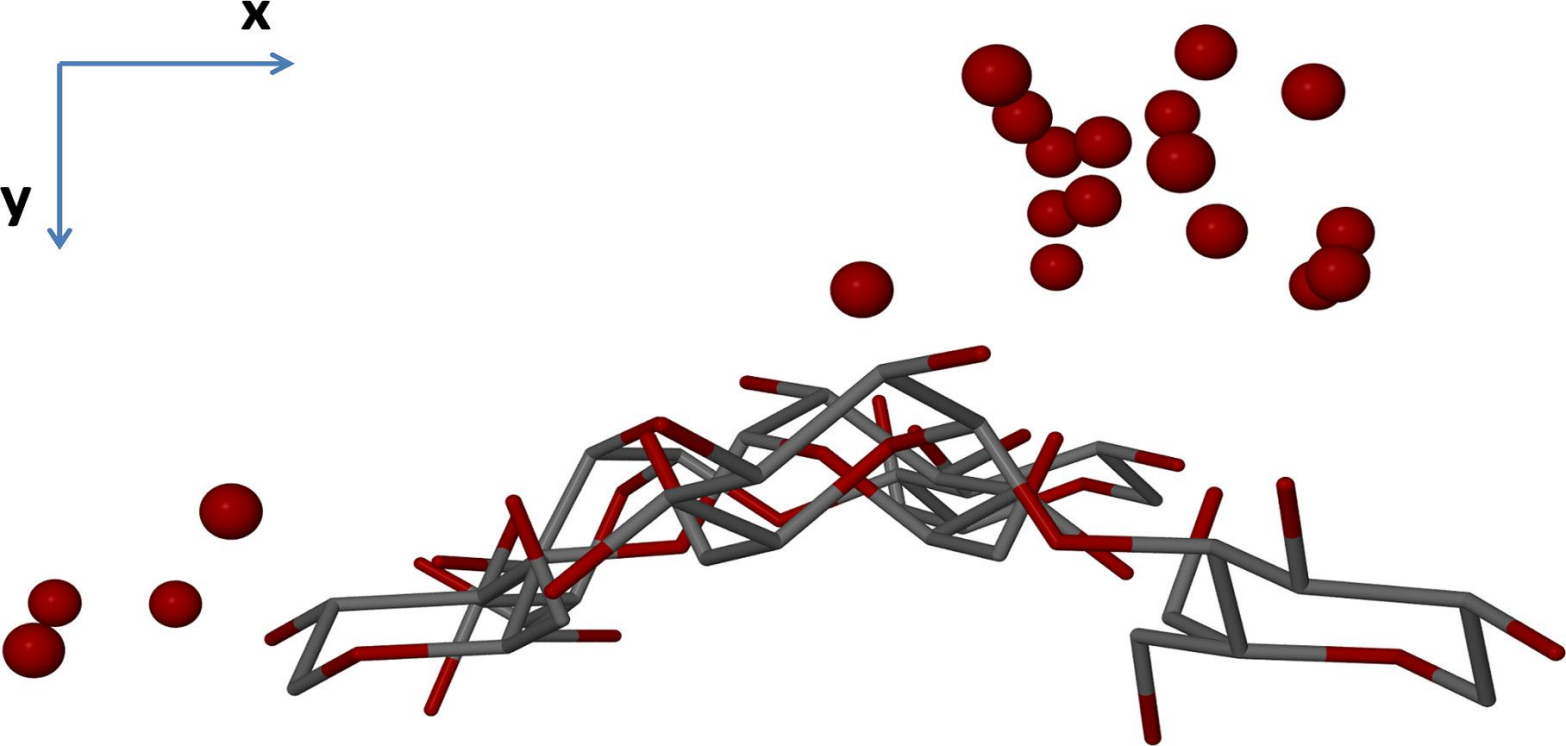
The host atoms and water oxygen atoms of the ASU viewed down the *c*-axis, showing that the water molecule sites are all external to the cavities of the γ-CD molecules.

With reference to γ-CD·PRO, the asymmetric unit (ASU) comprises three pairs of glucose rings that generate three complete γ-CD molecules when rotation of these units around the four-fold axis parallel to the *c*-axis is applied (Figure 9). The level of disorder in the host molecules was found to be minimal, only two of the hydroxymethyl groups being disordered over two positions. This is in contrast to the guest molecules which are instead severely disordered and consequently not observable. Water oxygen atoms were identified over 20 sites (as previously mentioned) and it is evident that these sites are all external to the γ-CD molecule cavities (Figure 10). Hydrogen atoms were not placed on the water oxygen atoms, as there was no evidence for them in the difference Fourier map.

**Figure 11:**
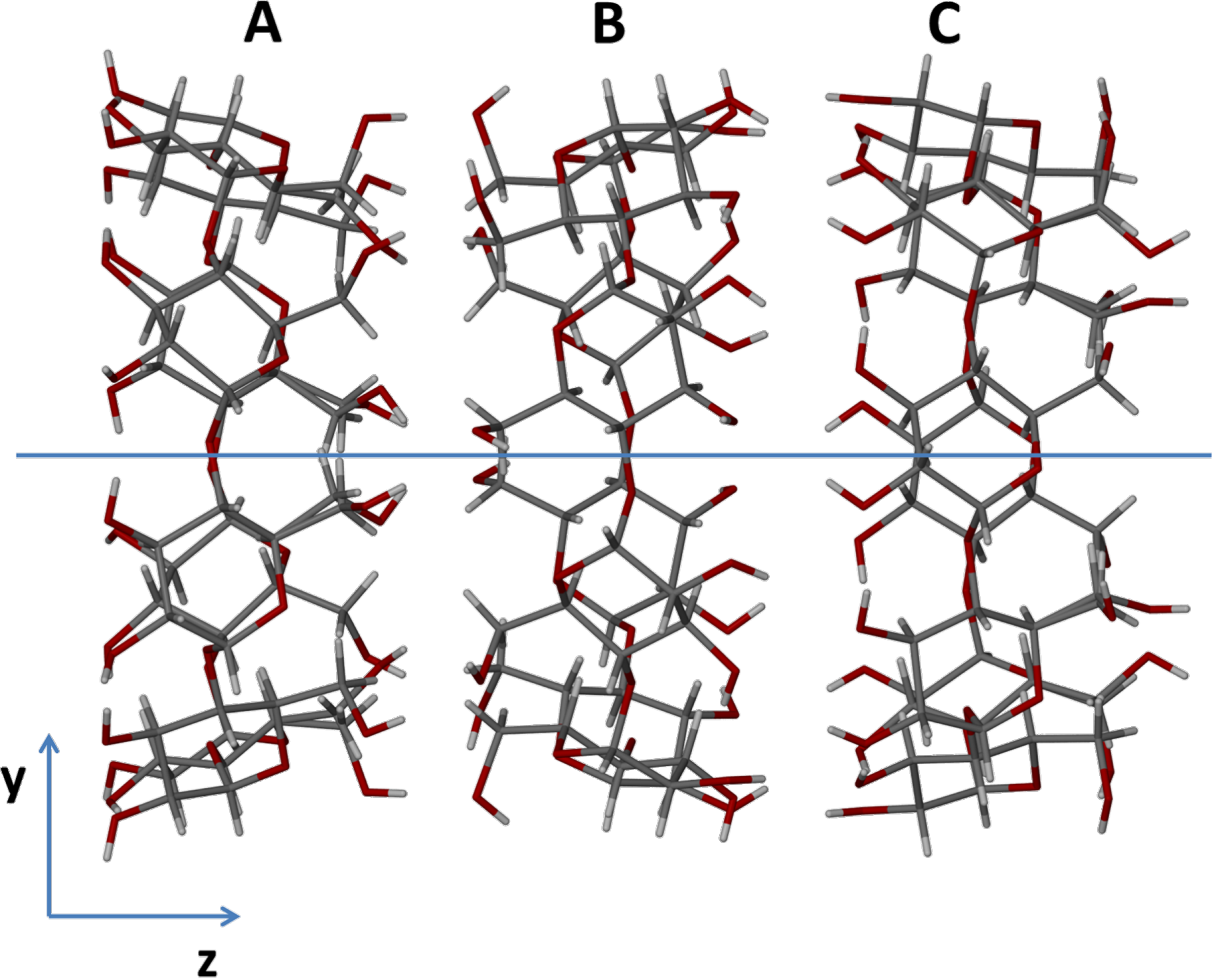
The distinct packing arrangement of the repeat unit of the host molecules in γ-CD·PRO. The four-fold axis (blue line) runs through the centre of the channel, parallel to the *c*-axis. The water molecules are omitted for clarity.

Three independent γ-CD molecules (A, B, C) comprise the major structural motif in the γ-CD·PRO crystal, and they stack to form infinite columns, arranging themselves in a head-to-tail motif between molecules C and A, a tail-to-tail motif between molecules B and C, and a head-to-head motif between molecules A and B (Figure 11) [46]. At each of the C–A, B–C and A–B interfaces extensive O–H···O hydrogen bonding takes place. This is a characteristic structural arrangement that has been observed in all γ-CD inclusion complexes crystallizing in the space group *P*42_1_2 [41].

**Figure 12:**
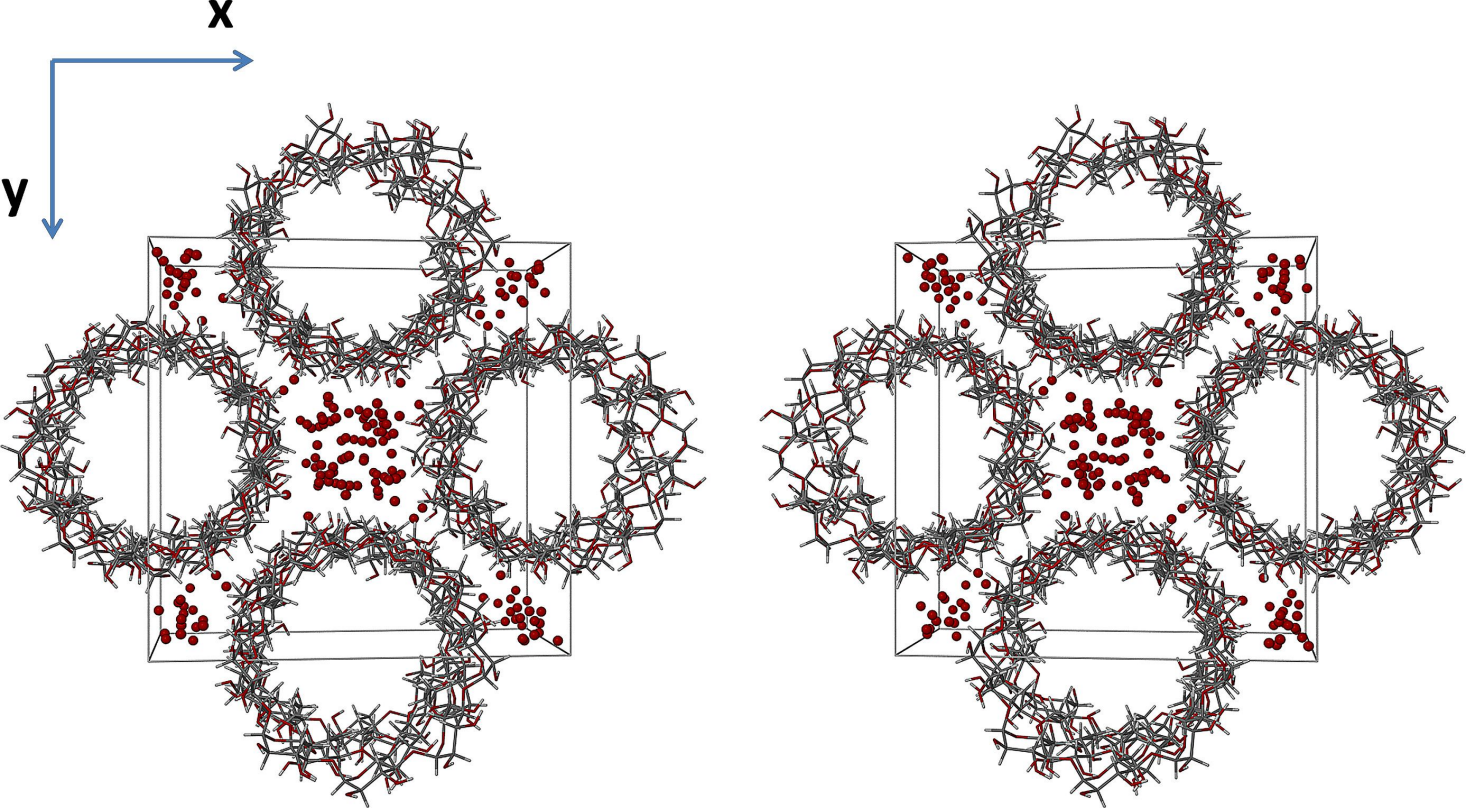
A stereoscopic view of γ-CD·PRO viewed down the *c*-axis, which displays the infinite channel packing arrangement. The water molecules (red spheres) occupy the interstitial spaces between the columns of stacked complex units.

Water molecules similarly occupy the interstitial spaces between the columns (Figure 12, viewed down the c-axis) and are thus also responsible for reinforcing the assembly of γ-CD complex columnar units via water–water and water–host hydrogen bonds. It should be noted that, since PXRD analyses indicated that the γ-CD·PRO and γ-CD·BES crystal structures are isostructural, all of the salient structural features illustrated above and below for the γ-CD·PRO crystal are common to the γ-CD·BES crystal, which was not amenable to single crystal X-ray analysis.

### Solubility analysis

Solubility determinations for the four native CD inclusion complexes (β-CD·BES, β-CD·PRO, γ-CD·BES and γ-CD·PRO) were performed in duplicate simultaneously. The intrinsic aqueous solubilities of 17β-estradiol and progesterone are 3.6 × 10^−3^ mg·cm^−3^ (at 27 °C) and 8.81 × 10^−3^ mg·cm^−3^ (at 25 °C), respectively [6,8], and the results obtained (Table 3) indicated that the CD inclusion of 17β-estradiol and progesterone did indeed enhance the aqueous solubility of both APIs significantly, most prominently in the cases of β-CD·BES and γ-CD·PRO. Improvement in the aqueous solubilities of PRO through its complexation with β-CD and γ-CD reported earlier [33] is consistent with the data for these complexes in Table 3.

**Table 3:** Aqueous solubility data for the β-CD·BES, β-CD·PRO, γ-CD·BES, and γ-CD·PRO inclusion complexes at 27 °C.

Number	CD complex	Solubility range of API (mg·cm^−3^)	Approximate solubility enhancement ratio (S_API(CD)_/S_API_)^a^	Average S_API(CD)_/S_API_ ratio

1	β-CD·BES produced via kneading (experiment 1)	0.0743–0.0784	21.8	21.9 ± 0.1
2	β-CD·BES produced via kneading (experiment 2)	0.0731–0.0790	21.9	

3	β-CD·PRO produced via kneading (experiment 1)	0.0380–0.0511	5.8	5.6 ± 0.2
4	β-CD·PRO produced via kneading (experiment 2)	0.0366–0.0474	5.4	

5	γ-CD·BES produced via kneading (experiment 1)	0.0193–0.0288	8.0	8.0 ± 0.1
6	γ-CD·BES produced via kneading (experiment 2)	0.0206–0.0283	7.9	

7	γ-CD·PRO produced via kneading (experiment 1)	0.162–0.174	19.8	19.9 ± 0.1
8	γ-CD·PRO produced via kneading (experiment 2)	0.161–0.175	19.9	

^a^S_API(CD)_ is the aqueous solubility of the API in the form of a CD inclusion complex, and S_API_ is the aqueous solubility of the API.

## Conclusion

The reported crystal structures of β-CD·BES, β-CD·PRO and γ-CD·PRO represent the first single crystal X-ray structural determinations of CD inclusion complexes of 17β-estradiol and progesterone. Comprehensive PXRD analyses of the complexes prepared by kneading enabled prediction of their space groups based on their isostructurality with known reference complexes. Comparison of these experimental patterns with simulated PXRD patterns based on SCXRD data revealed that the single crystals obtained by co-precipitation were isostructural with their counterparts prepared by kneading. The occurrence of severe guest disorder in all three cases precluded detailed determination of the host–guest interactions. Despite this limitation, complete chemical formulae were determined for the ternary complexes listed above, as well as that of γ-CD·BES, by combining accurate host–guest stoichiometric ratios obtained from ^1^H NMR analyses with the respective crystal water contents derived from thermogravimetric analyses. The aqueous solubilities of the two APIs, 17β-estradiol and progesterone, were found to be significantly enhanced via their encapsulation by β-CD and γ-CD. It is notable that these measured solubilities are also superior to those of the commercially utilized formulations containing ethinylestradiol and micronized progesterone. Further investigation should include in vivo experimentation to assess the efficacy of the four CD inclusion complexes as the active components of alternative commercial solid formulations for potential therapeutic treatment. Such CD-based formulations could be administered orally, as the absorption of cyclodextrins in the gastrointestinal tract is negligible (usually less than 4%) and the CDs are thus considered practically non-toxic [1–2]. Another advantageous feature of the four CD inclusion complexes investigated in this study is their ability to be synthesised rapidly via an efficient mechanochemical process, namely kneading of their respective host–guest mixtures, with water as the liquid medium. Furthermore, the proven significant API solubility enhancements reported here should result in less of the active ingredient being required in each dosage, thus rendering these CD inclusion complexes potentially financially attractive for pharmaceutical applications.

## Experimental

### Materials

17β-Estradiol (C_18_H_24_O_2,_) with purity >98% and progesterone (C_21_H_30_O_2_) with purity >98% were purchased from Sigma-Aldrich Chemie GmbH (Steinheim, Germany). β-Cyclodextrin (β-CD; C_42_H_70_O_35_) with purity >98% and water content 13.7(1)% (*n* = 2) was purchased from Cyclolab (Budapest, Hungary), while γ-cyclodextrin (γ-CD; C_48_H_80_O_40_) with purity >98% and water content 7.6(1)% (*n* = 2) was purchased from Wacker Biosolutions (Halle, Germany).

### CD complex preparation

**Kneading experiments:** Specific stoichiometric amounts of CD and API were utilized in the following host–guest ratios: β-CD·BES (2:1), β-CD·PRO (2:1), γ-CD·BES (1:1), γ-CD·PRO (3:2). The two components were placed in a mortar with a small amount of Milli-Q^®^ water [47], and the mixture was kneaded into a paste with a pestle for 40 minutes. Small increments (0.3 cm^3^) of water were added where necessary. These samples were used for the solubility determinations.

**Single-crystal preparation:** Initially, 35.24 mg (0.0310 mmol) of pure β-CD was dissolved in 3 cm^3^ water for the β-CD·BES system, and 30.53 mg (0.0269 mmol) of pure β-CD was dissolved in 15 cm^3^ water for the β-CD·PRO system. These CD solutions were stirred at 70 °C and the APIs were added in small increments over a 10 minute period to the respective CD solutions. 4.9 mg (0.0180 mmol) of pure BES and 4.9 mg (0.0156 mmol) of pure PRO were added, respectively. These amounts correspond to host–guest ratios of 1.72:1 and 1.72:1, respectively. Almost complete dissolution was attained after 15 minutes of stirring at 70 °C, but a very slight turbidity was still observed in the β-CD·BES solution. The solutions were left to stir for an additional 3 hours (to allow for the complex equilibrium to be achieved), after which they were filtered and prepared for slow cooling in a Dewar flask. Crystals appeared after 3 days and were shown to have 2:1 host/guest stoichiometry by ^1^H NMR spectroscopy.

Single-crystal preparation for both γ-CD·BES and γ-CD·PRO utilized a large excess of γ-CD in order to obtain crystalline inclusion complexes with 1:1 and 3:2 stoichiometric ratios, respectively. Approximately 100 mg (0.0771 mmol) of pure γ-CD was used for both systems, and the masses of the pure APIs were both 4.9 mg (0.0180 mmol for 17β-estradiol and 0.0156 mmol for progesterone). This corresponded to host–guest ratios of 4.28:1 and 4.94:1, respectively. The two weighed samples of γ-CD were dissolved in water in separate vials (3 cm^3^ for the γ-CD·PRO system and 2.5 cm^3^ for the γ-CD·BES system) and stirred vigorously at 70 °C. The APIs were added fairly slowly to their respective vials over a period of about 5 minutes and the solutions were left to stir for a further 3 hours and were subsequently filtered and prepared for slow cooling. Crystals appeared after 3 days, and it should be noted that complete evaporation of the mother liquors must be avoided in order to prevent the excess γ-CD subsequently precipitating. Subsequently, ^1^H NMR spectroscopy revealed host/guest stoichiometries of 1:1 for γ-CD·BES and 3:2 for γ-CD·PRO.

### Powder X-ray diffraction (PXRD)

PXRD patterns were recorded at 23 °C for each reagent as well as the products formed from kneading and co-precipitation. PXRD analyses were performed on a Bruker D2 Phaser desktop powder diffractometer (Billerica, Massachusetts, U.S.A.) with Cu Kα_1_ radiation (λ = 1.5406 Å) with the X-ray generator set at 30 kV and 10 mA. All samples were lightly ground (to minimise the effects of preferred orientation) and placed on a silicon zero-background sample holder. The scanning range was 4^o^ – 40^o^ 2θ with a step size of 0.0164^o^ and a primary beam path slit of 0.6 mm.

### ^1^H NMR spectroscopy

Solution-state ^1^H NMR analyses were performed to quantify the stoichiometric ratios of the CD inclusion complexes, and all of the crystalline samples obtained by co-precipitation were dissolved in deuterated dimethyl sulfoxide (DMSO-*d*_6_) at 23 °C. These analyses were performed on a Bruker Ultrashield 400 Plus Spectrometer (Billerica, Massachusetts, U.S.A.) and the program MestReNova was used to analyse the resulting data [48].

### Thermal analysis

**Hot stage microscopy (HSM):** The crystals were immersed in a small amount of silicone oil and heated at a constant rate of 10 K·min^−1^ until decomposition. The HSM experiment was viewed through a Nikon SMZ-10 stereoscopic microscope fitted with a Linkam THM600 hot stage and a Linkam TP92 temperature control unit. The images were captured by a real-time Sony Digital Hyper HAD colour video at selected temperatures. The captured images were viewed with the Soft Imaging Program AnalySIS [49].

**Thermogravimetric analysis (TGA):** TGA analyses were performed on the TA-Q500 (Texas Instruments) with Universal Analysis 2000 software. Sample preparation involved rapidly removing the crystals from their mother liquor and subsequently lightly pressing them between a filter paper to dry their surfaces, and the TGA experiment commenced immediately after the mother liquor was removed. Masses between 0.7 mg and 2.0 mg were used for the analyses, which were performed either in duplicate or triplicate. The analyses took place in open pans under dry nitrogen gas with a constant flow rate of 60 cm^3^·min^−1^ and the samples were heated to a maximum temperature of 400 °C at a constant rate of 10 K·min^−1^.

**Differential scanning calorimetry (DSC):** DSC analyses were performed on a DSC XP-10 instrument (Surface Solutions GmbH), and the data analysed with TRIOS software [50]. Sample preparation entailed drying the crystal surfaces from the mother liquor and placing the crystals in a crimped aluminium pan with two venting holes. A constant rate of 10 K·min^−1^ was used to heat the samples (mass range 1–2 mg) under dry N_2_ purge gas with a flux of 60 cm^3^·min^−1^. All DSC experiments were terminated prior to the decomposition of each sample.

### Solubility analysis by gravimetric increments

A gravimetric solubility approach was used involving the addition of small, accurately pre-weighed incremental amounts of a given CD inclusion complex (total mass approximately 30 mg each) into a vial containing 3 cm^3^ of water. A visual estimation of the solubility was established after the final suspension was stirred for 72 hours at 27 °C. This procedure ensured that a very narrow solubility range was spanned by the penultimate and final incremental CD inclusion complex additions, the final amount resulting in saturation of the solution. The apparatus involved a Radleys Standard stirring hotplate with a 2.5 cm high vial supporting stand attached to it. The vials were placed on the stand in a circle at a radius of 4 cm from the centre, and the solutions were stirred at a rate of 250 rpm for 72 hours.

### Single crystal X-ray diffraction (SCXRD) analysis

Unit cell determinations and data-collections were performed on a Bruker KAPPA APEX II DUO single-crystal X-ray diffractometer (Madison Wisconsin, U.S.A.) and a Bruker D8 VENTURE SCXRD single-crystal X-ray diffractometer (Madison Wisconsin, U.S.A.). The crystals were coated in Paratone N oil [51]. The unit cell determinations were initially performed at room temperature and thereafter reconfirmed following cooling to 100(2) K in a nitrogen vapour stream using an Oxford Cryostream cooler (Oxford Cryosystems Ltd, Oxford, U.K.). The data-collection was then performed and the collected intensity datasets were read into the program XPREP [52]. The structures were solved using isomorphous replacement, and the water oxygen atoms were refined anisotropically if they possessed full site-occupancy. No water hydrogen atoms were included in the models. Thereafter, the structures were refined by full-matrix least-squares techniques with SHELXL-97 [53], implemented in the X-SEED [54] interface.

## Supporting Information

File 1 PXRD patterns, ^1^H NMR data, thermal data for hot stage microscopy (HSM), variable temperature powder X-ray diffraction (VTPXRD) patterns, TGA, dTGA and DSC analytical data, details of electron counts derived from the Squeeze procedure, and geometrical parameters of the β-CD host molecules.
