# Supplementary material for: Inclusion complexes of the steroid hormones 17β-estradiol and progesterone with β- and γ-cyclodextrin hosts: syntheses, X-ray structures, thermal analyses and API solubility enhancements
Source: Beilstein J Org Chem. 2022 Dec 22;18:1749–62. doi: 10.3762/bjoc.18.184 (PMC9795861; doi:10.3762/bjoc.18.184)
Supplement: File 1 — PXRD patterns, 1H NMR data, thermal data for hot stage microscopy (HSM), variable temperature powder X-ray diffraction (VTPXRD) patterns, TGA, dTGA and DSC analytical data, details of electron counts derived from the Squeeze procedure, and geometrical parameters of the β-CD host molecules. [file Beilstein_J_Org_Chem-18-1749-s001.pdf]

BEILSTEIN JOURNAL OF ORGANIC CHEMISTRY

Supporting Information

for

Inclusion complexes of the steroid hormones 17β-estradiol and progesterone with β- and γ-cyclodextrin hosts: syntheses, X-ray structures, thermal analyses and API solubility enhancements

Alexios I. Vicatos, Zakiena Hoossen and Mino R. Caira

Beilstein J. Org. Chem. **2022,** *18,* 1749–1762. doi:10.3762/bjoc.18.184

PXRD patterns, ^{1}H NMR data, thermal data for hot stage microscopy (HSM), variable temperature powder X-ray diffraction (VTPXRD) patterns, TGA, dTGA and DSC analytical data, details of electron counts derived from the Squeeze procedure, and geometrical parameters of the β-CD host molecules

License and Terms: This is a supporting information file under the terms of the Creative Commons Attribution License (https://creativecommons.org/licenses/by/4.0). Please note that the reuse, redistribution and reproduction in particular requires that the author(s) and source are credited and that individual graphics may be subject to special legal provisions.
The license is subject to the *Beilstein Journal of Organic Chemistry* terms and conditions: (https://www.beilstein-journals.org/bjoc/terms)

Contents

Section 1: PXRD patterns………………………………………………………………………………………………… p. S3

Fig. S1 The PXRD patterns of the β-CD·BES complex produced via kneading (2:1), an isostructural β-CD complex crystallizing in the space group *C*2 (refcode: DOCYID), and the β-CD·BES complex produced via co-precipitation (2:1).

Fig. S2 The PXRD patterns of the γ-CD·BES complex produced *via* kneading (1:1), an isostructural γ-CD complex crystallizing in the space group $P42_12$ (refcode: DOCYID), and the γ-CD·BES complex produced *via* co-precipitation (1:1).

Section 2: Supplementary ^{1}H NMR data…………………………………………………………………………………p. S4

Fig. S3 Molecular structures of β-estradiol (left), progesterone (centre), and β-CD (right), with specific molecular fragments labelled for the ^{1}H NMR analysis. Adapted from Guedes et al.[1]

Fig. S4 The ^{1}H NMR spectrum of β-CD·BES.

Table S1. The ^{1}H NMR spectral analysis and stoichiometric assignments for β-CD·BES.

Fig. S5 The ^{1}H NMR spectrum of β-CD·PRO.

Table S2. The ^{1}H NMR spectral analysis and stoichiometric assignments for β-CD·PRO.

Fig. S6 Molecular structures of β-estradiol (left), progesterone (centre) and γ-CD (right). Specific atoms have been labelled for the ^{1}H NMR analyses. Adapted from Guedes et al.[1]

Fig. S7 The ^{1}H NMR spectrum of γ-CD·BES.

Table S3. The ^{1}H NMR spectral analysis and stoichiometric assignments of γ-CD·BES.

Fig. S8 The ^{1}H NMR spectrum of γ-CD·PRO.

Table S4. The ^{1}H NMR spectral analysis and stoichiometric assignments of γ-CD·PRO.

Section 3: Supplementary thermal data for the hot stage microscopy (HSM) analysis……………p. S9

Fig. S9 The series of HSM micrographs for β-CD·BES.

Fig. S10 The series of HSM micrographs for β-CD·PRO.

Fig. S11 The series of HSM micrographs for γ-CD·BES.

Fig. S12 The series of HSM micrographs for γ-CD·PRO.

Section 4: Supplementary data for the variable temperature powder X-ray diffraction (VTPXRD) analysis………p. S13

Fig. S13 The variable temperature PXRD patterns of β-CD·BES over the temperature range from 25 °C to 235 °C. The numbers in the key represent the temperature in °C. The peak at the angular position of 7.0° is an artefact of the variable temperature PXRD stage. No significant peaks were observed at angular positions greater than 30.0°.

Fig. S14 The variable temperature PXRD patterns of β-CD·PRO over the temperature range from 25 °C to 200 °C. The numbers in the key represent the temperature in °C. The peak at the angular position of 7.0° is an artefact of the variable temperature PXRD stage. No significant peaks were observed at angular positions greater than 30.0°.

Section 5: Supplementary data for the TGA, dTGA and DSC analyses.......................................p. S15

Fig. S15 A representative DSC curve (*n* = 2) of β-CD·BES, with the respective TGA curve (*n* = 3).

Fig. S16 A representative DSC curve (*n* = 2) of β-CD·PRO, with the respective TGA curve (*n* = 2).

Fig. S17 A representative DSC curve of γ-CD·BES (*n* = 2), with the respective TGA curve (*n* = 2).

Fig. S18 A representative DSC curve of γ-CD·PRO (*n* = 3), with the respective TGA curve (*n* = 3).

Fig. S19 Representative TGA and dTGA curves for β-CD·BES (*n* = 3).

Fig. S20 Representative TGA and dTGA curves for β-CD·PRO (*n* = 2).

Fig. S21 Representative TGA and dTGA curves of γ-CD·BES (*n* = 2).

Fig. S22 Representative TGA and dTGA curves of γ-CD·PRO (*n* = 3).

Section 6: Estimating the host-guest stoichiometries of the β-CD·BES, β-CD·PRO and γ-CD·PRO complexes from electron counts derived from the Squeeze procedure......................................p. S21

Fig. S23 The calculated PXRD patterns of β-CD·BES, β-CD·PRO and γ-CD·PRO as well as the PXRD patterns of β-CD·BES, β-CD·PRO and γ-CD·PRO prepared *via* co-precipitation.

Section 7: The geometrical parameters of the β-CD host molecules...p. S22

Table S5: The geometrical parameters of the host molecule in the complex β-CD·BES.

Fig. S24 The numbering scheme of the β-CD·BES glucose units.

Table S6: The geometrical parameters of the host molecule in the complex β-CD·PRO.

Fig. S25 The numbering scheme of the β-CD·PRO glucose units.

REFERENCES...p. S23

Section 1: PXRD patterns

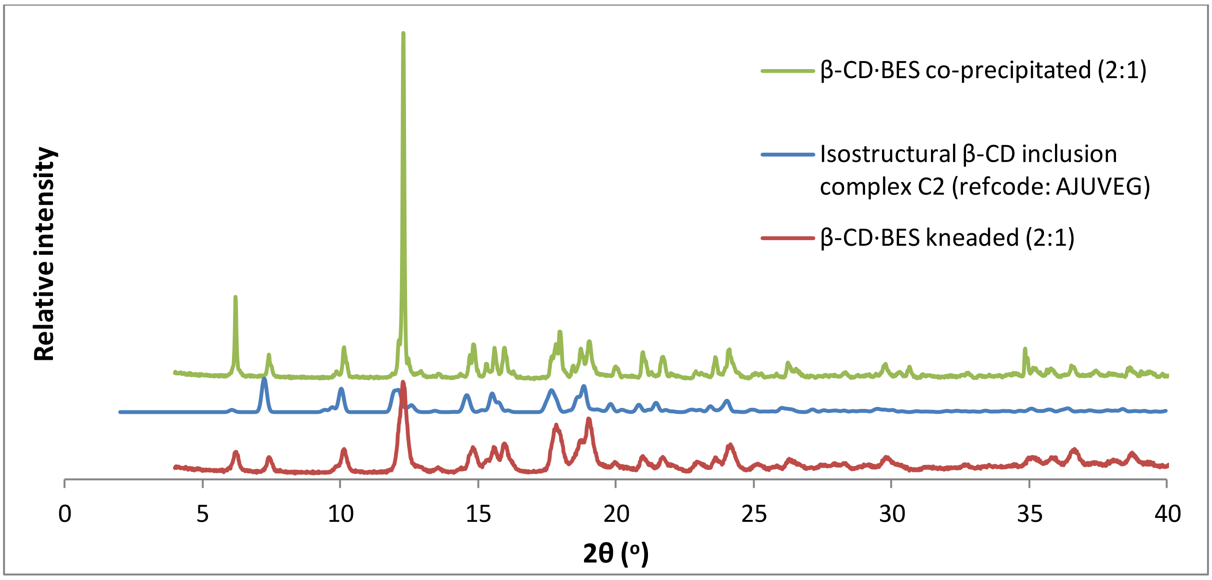

Fig. S1 The PXRD patterns of the β-CD·BES complex produced via kneading (2:1), an isostructural β-CD complex crystallizing in the space group *C*2 (refcode: DOCYID), and the β-CD·BES complex produced *via* co-precipitation (2:1).

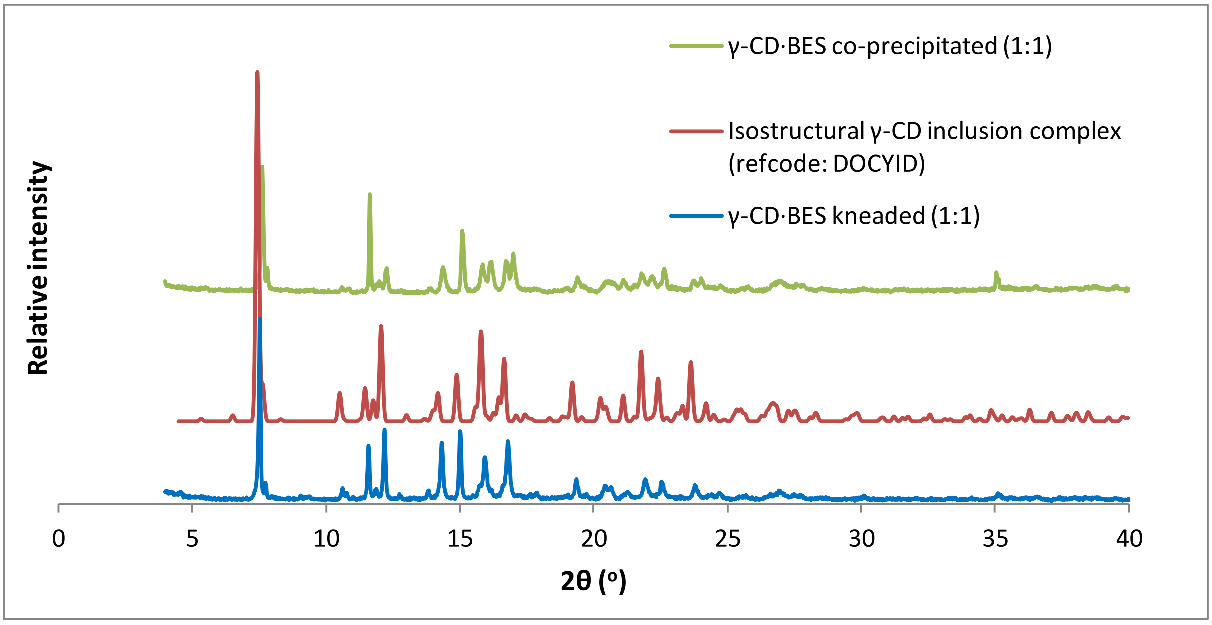

Fig. S2 The PXRD patterns of the γ-CD·BES complex produced via kneading (1:1), an isostructural γ-CD complex crystallizing in the space group $P42_12$ (refcode: DOCYID), and the γ-CD·BES complex produced *via* co-precipitation (1:1).

Section 2: Supplementary ^{1}H NMR data

<u>The ^{1}H NMR analyses of β-CD·BES and β-CD·PRO.</u>

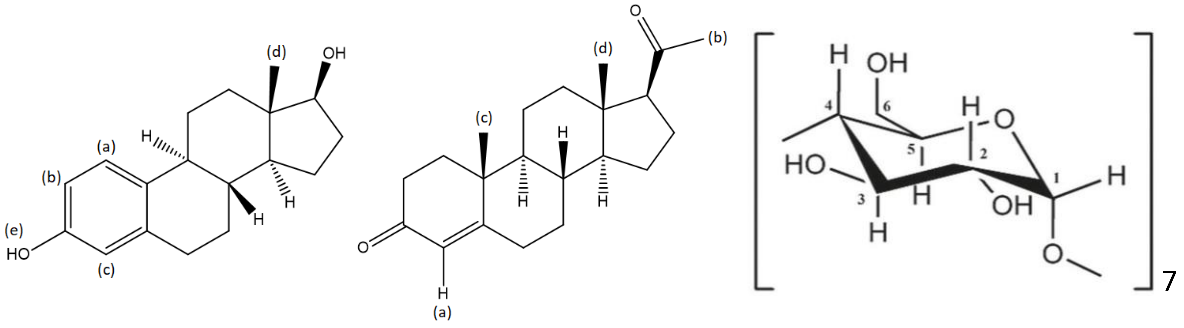

Fig.S3 Molecular structures of β-estradiol (left), progesterone (centre), and β-CD (right), with specific molecular fragments labelled for the ^{1}H NMR analysis. Adapted from Guedes et al.[1]

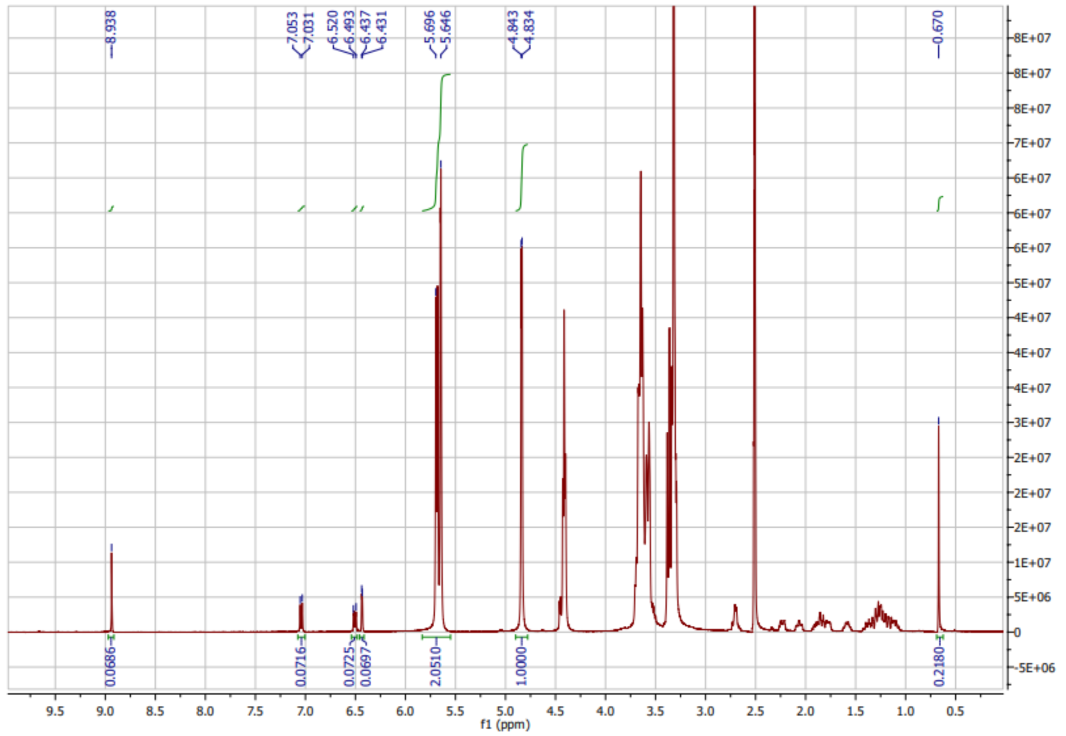

Fig.S4 The ^{1}H NMR spectrum of β-CD·BES.

Table S1. The ^{1}H NMR spectral analysis and stoichiometric assignments for β-CD·BES.

Assignment	δ (ppm)	Integration	Multiplicity	Proton representation (per molecule)	Stoichiometric ratio	Stoichiometric ratio (integer)
O<u>H</u> (e) (BES)	8.938	0.0686	singlet	1H	0.48	1
C<u>H</u> (a) aromatic (BES)	7.031 – 7.053	0.0716	doublet	1H	0.50	1
C<u>H</u> (b) aromatic (BES)	6.493 – 6.520	0.0725	doublet of doublets	1H	0.51	1
C<u>H</u> (c) aromatic (BES)	6.431 – 6.437	0.0697	doublet	1H	0.49	1
O<u>H</u>-2 and O<u>H</u>-3 (β-CD)	5.646 – 5.696	2.0510	two separate doublets	14H	1.03	2
C<u>H</u>-1 (β-CD)	4.834 – 4.843	1.0000*	doublet	7H	1.00	2
$C\underline{H_3}$ (d) methyl (BES)	0.670	0.2180	singlet	3H	0.51	1

*reference integral

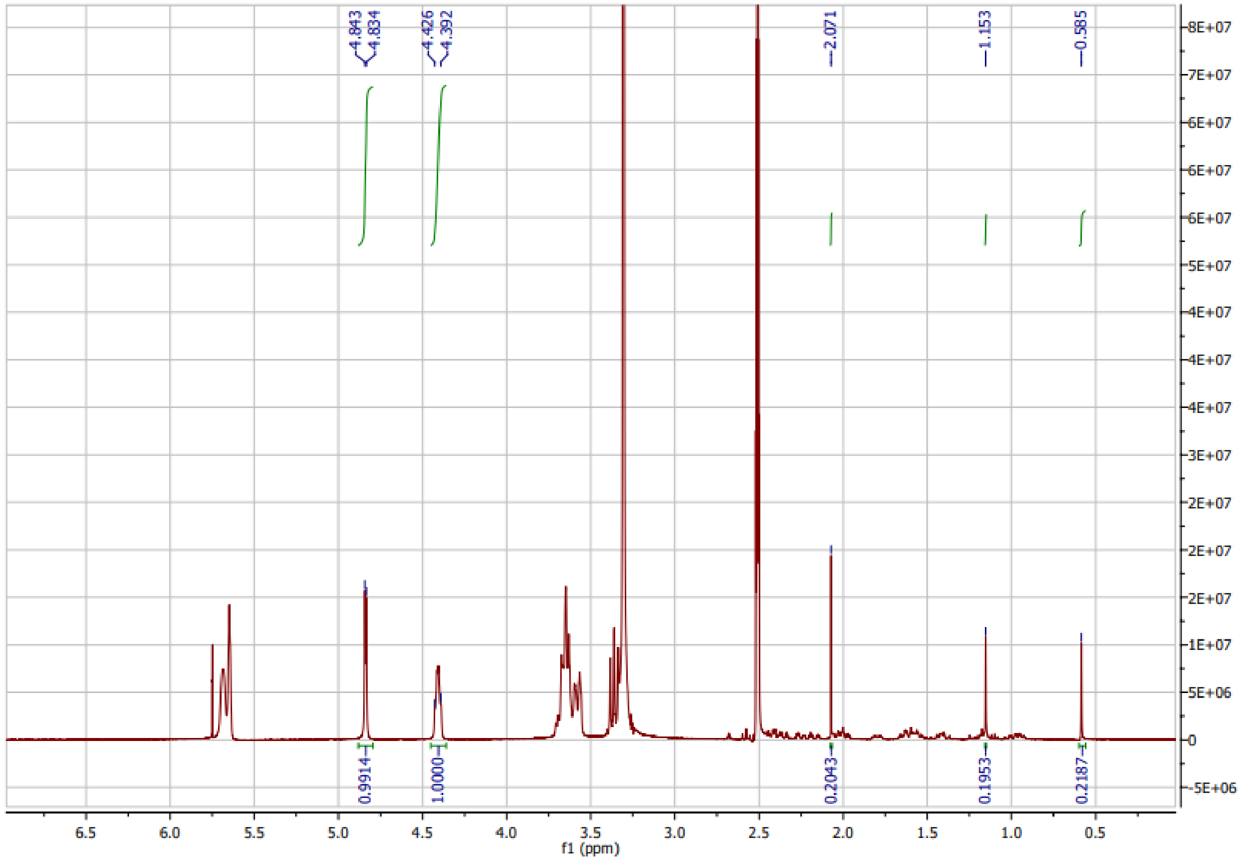

Fig.S5 The ^{1}H NMR spectrum of β-CD·PRO.

Table S2. The ^{1}H NMR spectral analysis and stoichiometric assignments for β-CD·PRO.

Assignment	δ (ppm)	Integration	Multiplicity	Proton representation (per molecule)	Stoichiometric ratio	Stoichiometric ratio (integer)
CH-1 (β-CD)	4.834 – 4.843	0.9914	doublet	7H	0.99	2
OH-6 (β-CD)	4.392 – 4.426	1.0000*	quartet	7H	1.00	2
CH_3 (a) methyl (PRO)	2.071	0.2043	singlet	3H	0.48	1
CH_3 (b) methyl (PRO)	1.153	0.1953	singlet	3H	0.46	1
CH_3 (c) methyl (PRO)	0.585	0.2187	singlet	3H	0.51	1

*reference integral

The ^{1}H NMR analyses of γ-CD·BES and γ-CD·PRO.

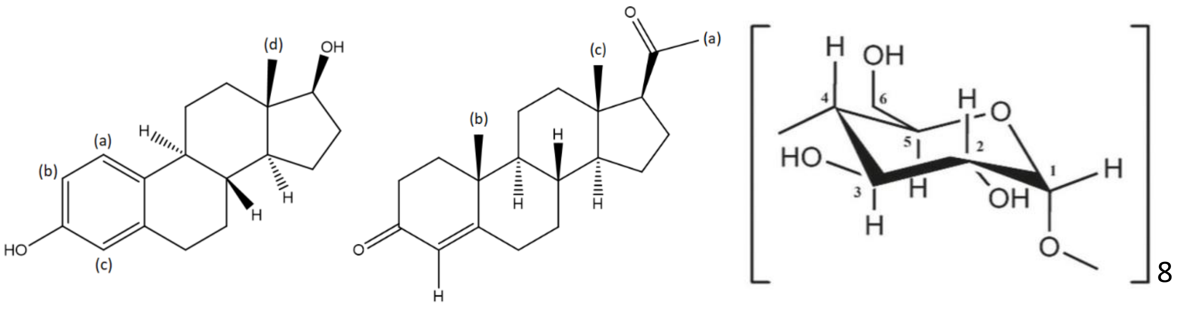

Fig.S6 Molecular structures of β-estradiol (left), progesterone (centre) and γ-CD (right). Specific atoms have been labelled for the ^{1}H NMR analyses. Adapted from Guedes *e*

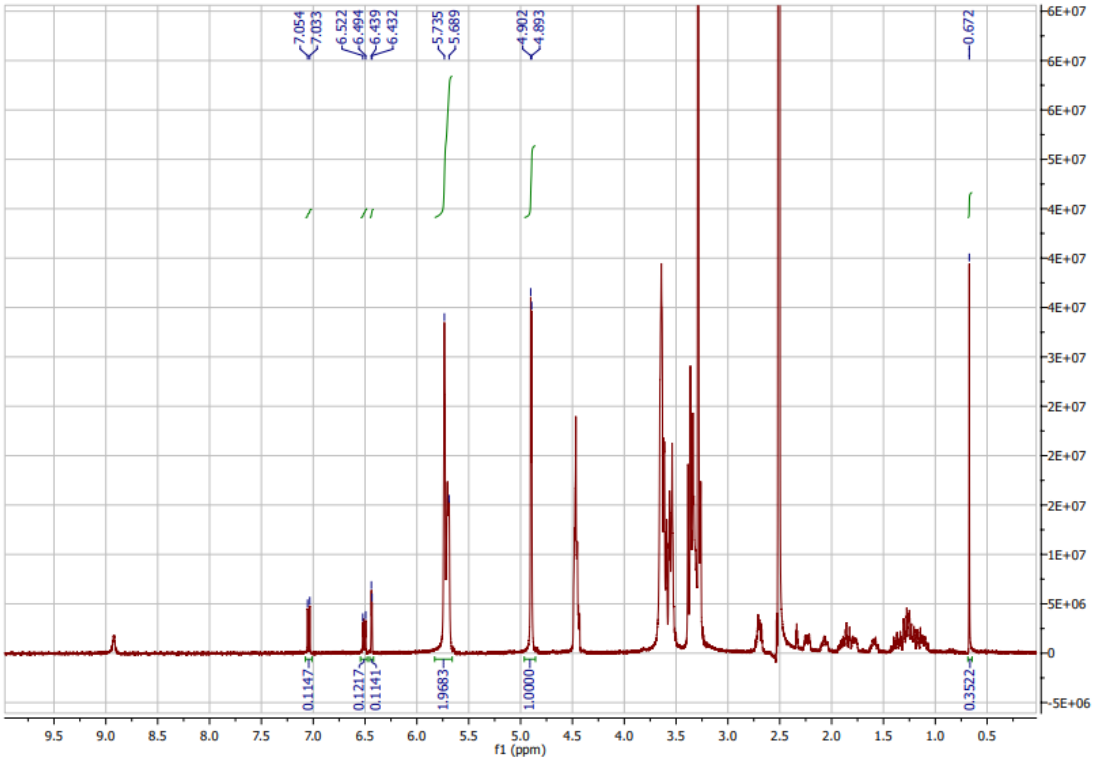

t al.[1]

Fig.S7 The ^{1}H NMR spectrum of γ-CD·BES.

Table S3. The ^{1}H NMR spectral analysis and stoichiometric assignments of γ-CD·BES.

Assignment	δ (ppm)	Integration	Multiplicity	Proton representation (per molecule)	Stoichiometric ratio	Stoichiometric ratio (integer)
CH (a) aromatic (BES)	7.033 – 7.054	0.1147	doublet	1H	0.92	1
CH (b) aromatic (BES)	6.494 – 6.522	0.1217	doublet of doublets	1H	0.97	1
CH (c) aromatic (BES)	6.432 – 6.439	0.1141	doublet	1H	0.91	1
OH-2 and OH-3 (γ-CD)	5.689 – 5.735	1.9683	Two separate doublets[1]	16H	0.98	1
CH-1 (γ-CD)	4.893 – 4.902	1.0000*	doublet	8H	1.00	1
CH_3 (d) methyl (BES)	0.672	0.3522	singlet	3H	0.94	1

*reference integral

[1] The ^{1}H NMR spectrum displays one doublet and a singlet with a shoulder. This shoulder would resolve into the second peak to become the other doublet at a higher resolution, or if more mass was available at the time of the experiment (which was the case with β-CD·BES).

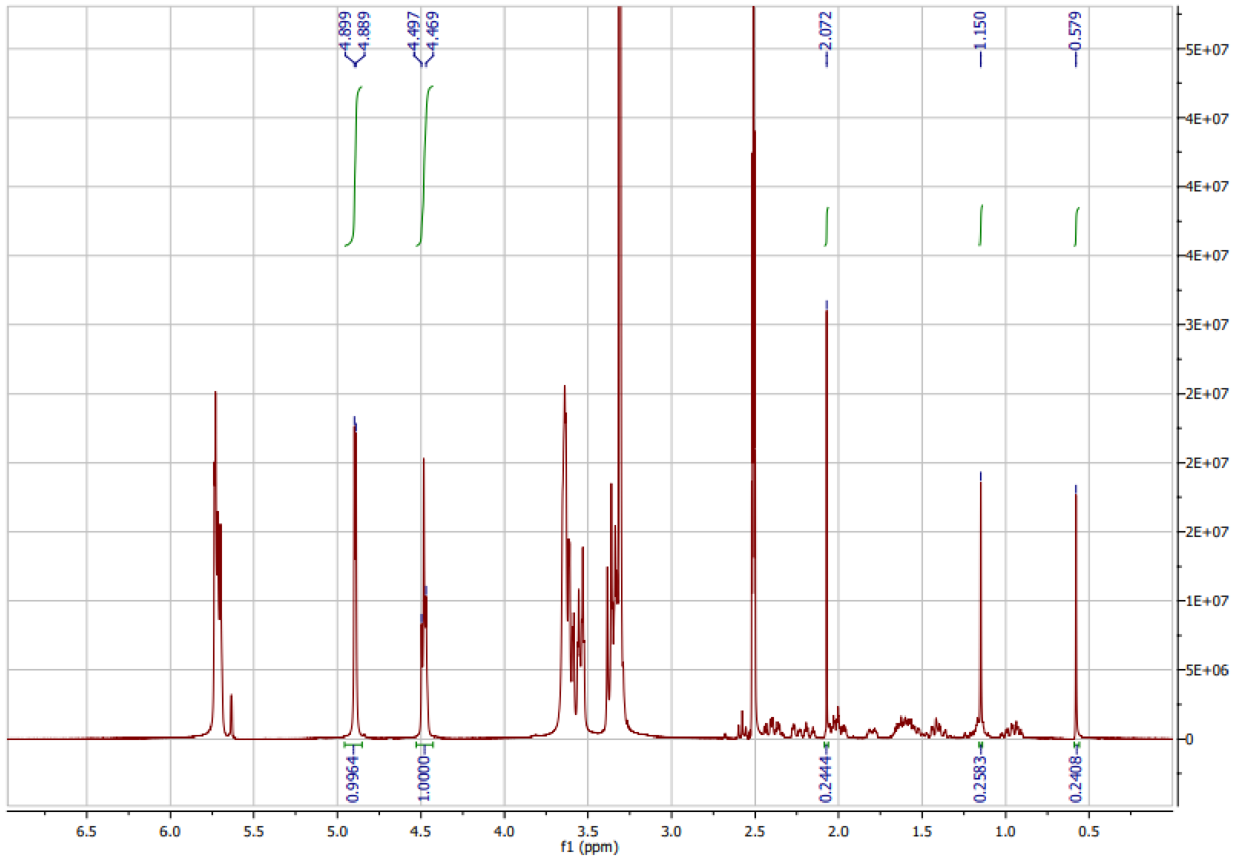

Fig.S8 The ^{1}H NMR spectrum of γ-CD·PRO.

Table S4. The ^{1}H NMR spectral analysis and stoichiometric assignments of γ-CD·PRO.

Assignment	δ (ppm)	Integration	Multiplicity	Proton representation (per molecule)	Stoichiometric ratio	Stoichiometric ratio (integer)
CH-1 (γ-CD)	4.889 – 4.899	0.9964	doublet	8H	1.00	3
OH-6 (γ-CD)	4.469 -4.497	1.0000*	triplet	8H	1.00	3
CH_3 (a) methyl (PRO)	2.072	0.2444	singlet	3H	0.65	2
CH_3 (b) methyl (PRO)	1.150	0.2583	singlet	3H	0.69	2
CH_3 (c) methyl (PRO)	0.579	0.2408	singlet	3H	0.64	2

*reference integral

Section 3: Supplementary thermal data for the hot stage microscopy (HSM) analysis.

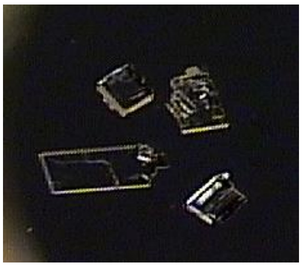

A: Initial appearance (25.5 °C).

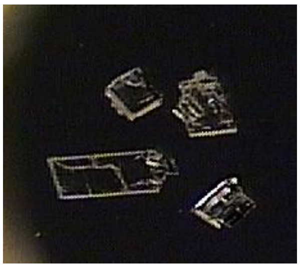

B: Onset of opacity owing to dehydration (57.4 °C).

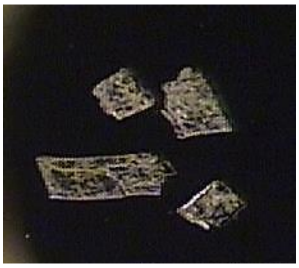

C: Increase in opacity, as a result of internal crystal fracturing owing to dehydration (70.1 °C).

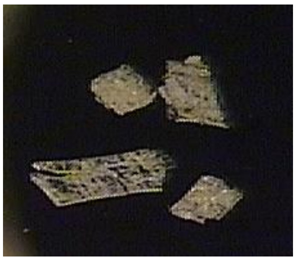

D: The crystals visually start to disintegrate owing to dehydration (80.7 °C).

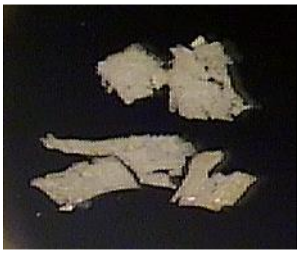

E: The crystals have ceased fracturing at 209.5 °C.

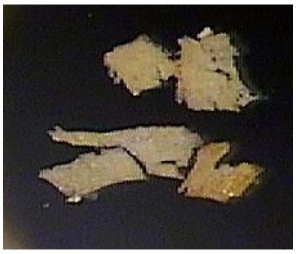

F: Onset of decomposition (255.5 °C).

Fig.S9 The series of HSM micrographs for β-CD·BES.

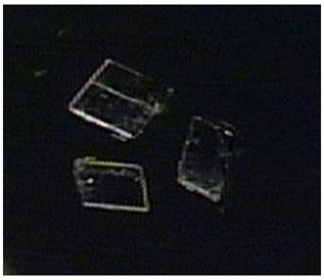

A: Initial appearance (22.5 °C).

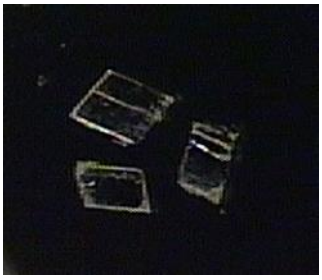

B: Onset of rapid crystal fracturing owing to dehydration (55.3 °C).

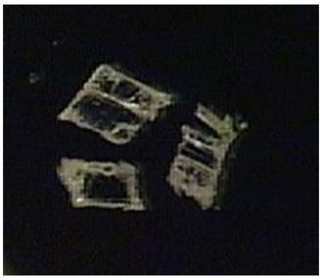

C: Rapid continuation of crystal fracturing owing to dehydration (63.3 °C).

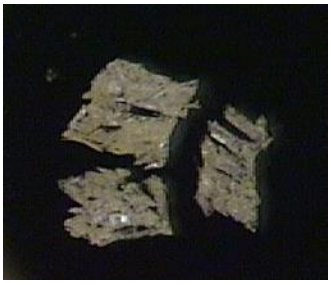

D: Rapid continuation of crystal disintegration owing to dehydration (73.9 °C).

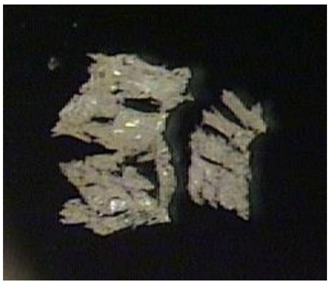

E: No observable changes other than the fragments drifting (79.7 °C).

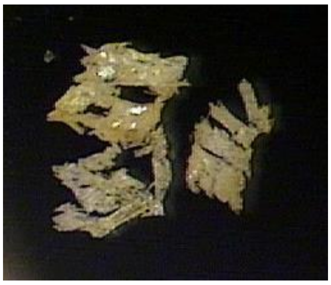

F: Onset of decomposition (292.7 °C).

Fig.S10 The series of HSM micrographs for β-CD·PRO.

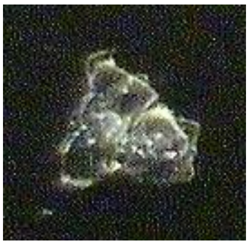

A: Initial appearance of γ-CD·BES (22.4 °C).

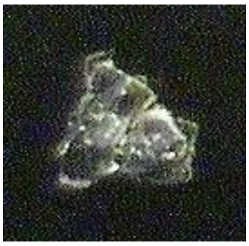

B: Hardly discernible onset of opacity owing to dehydration (29.4 °C).

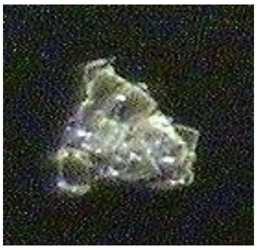

C: Continuation of opacity owing to dehydration (50.4 °C).

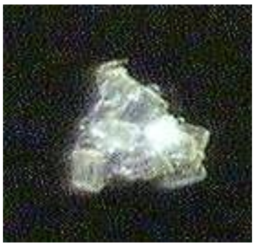

D: Initial level of opacity achieved owing to dehydration (88.2 °C).

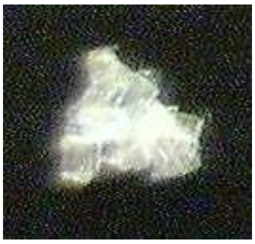

E: Maximum opacity achieved owing to dehydration (257.7 °C).

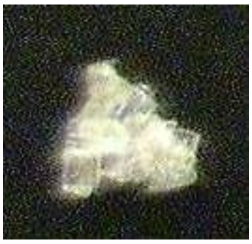

F: Onset of decomposition (262.5 °C).

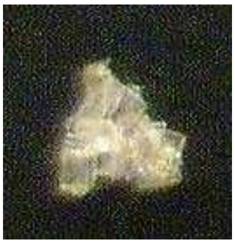

G: Continuation of decomposition (281.9 °C).

Fig.S11 The series of HSM micrographs for γ-CD·BES.

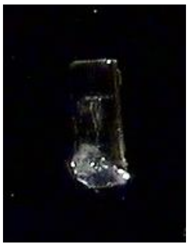

A: Initial appearance of γ-CD·PRO (23.7 °C).

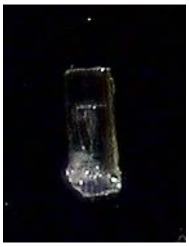

B: The onset of opacity owing to dehydration owing to dehydration (70.1 °C).

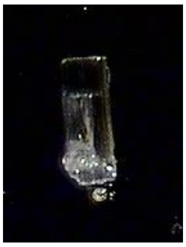

C: The onset of bubbling owing to dehydration (88.4 °C).

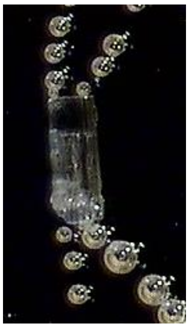

D: The maximum bubbling occurred (103.6 °C).

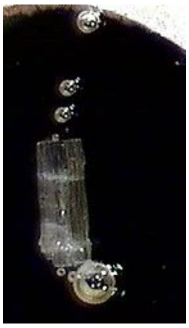

E: The bubbling lessened (145.5 °C).

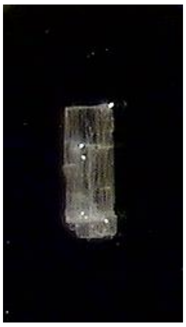

F: The bubbling terminated (193.4 °C).

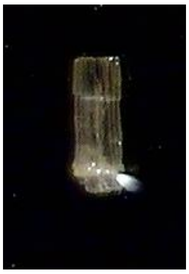

G: The onset of decomposition (263.4 °C).

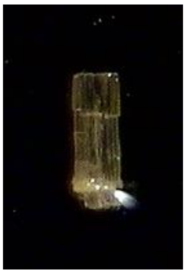

H: The continuation of decomposition (286.6 °C).

Fig.S12 The series of HSM micrographs for γ-CD·PRO.

Section 4: Supplementary data for the variable temperature powder X-ray diffraction (VTPXRD) analysis.

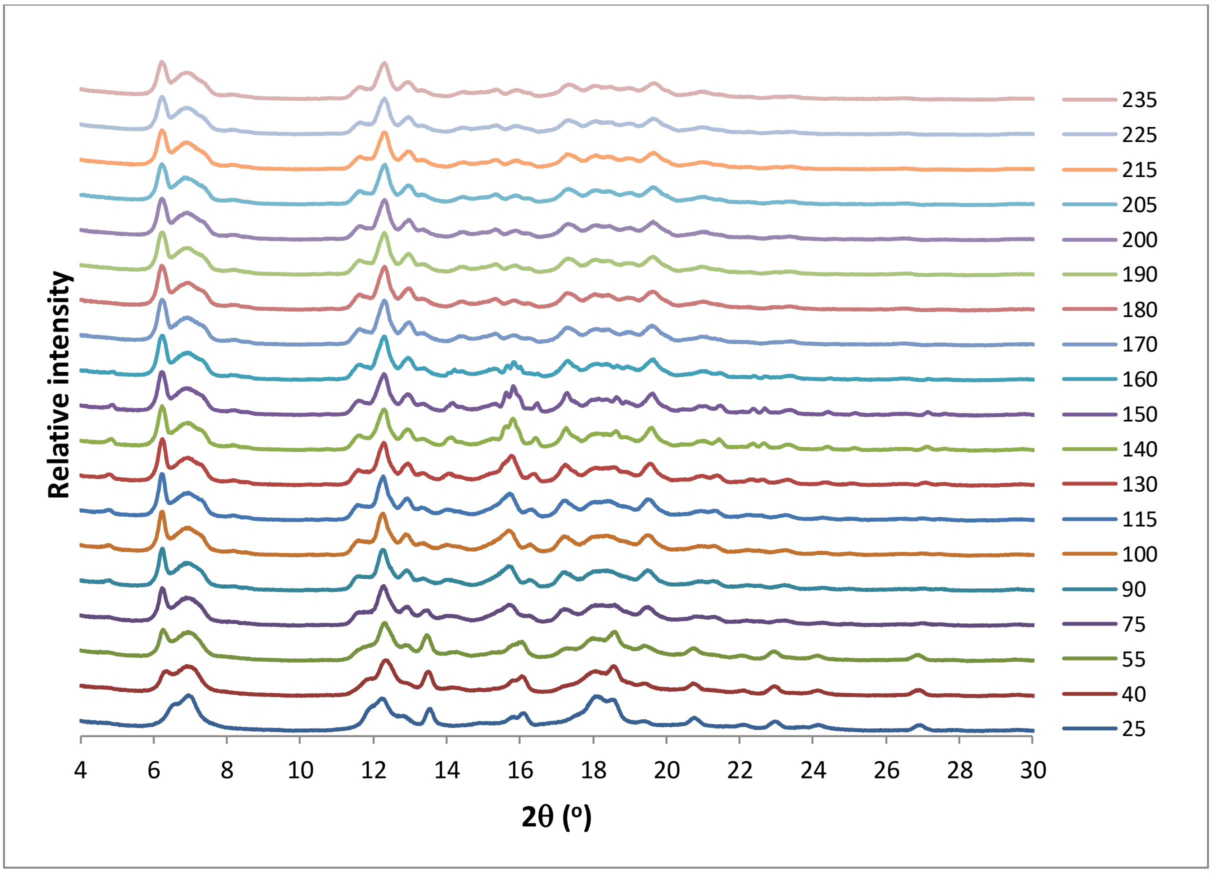

Fig.S13 The variable temperature PXRD patterns of β-CD·BES over the temperature range from 25 °C to 235 °C. The numbers in the key represent the temperature in °C. The peak at the angular position of 7.0° is an artefact of the variable temperature PXRD stage. No significant peaks were observed at angular positions greater than 30.0°.

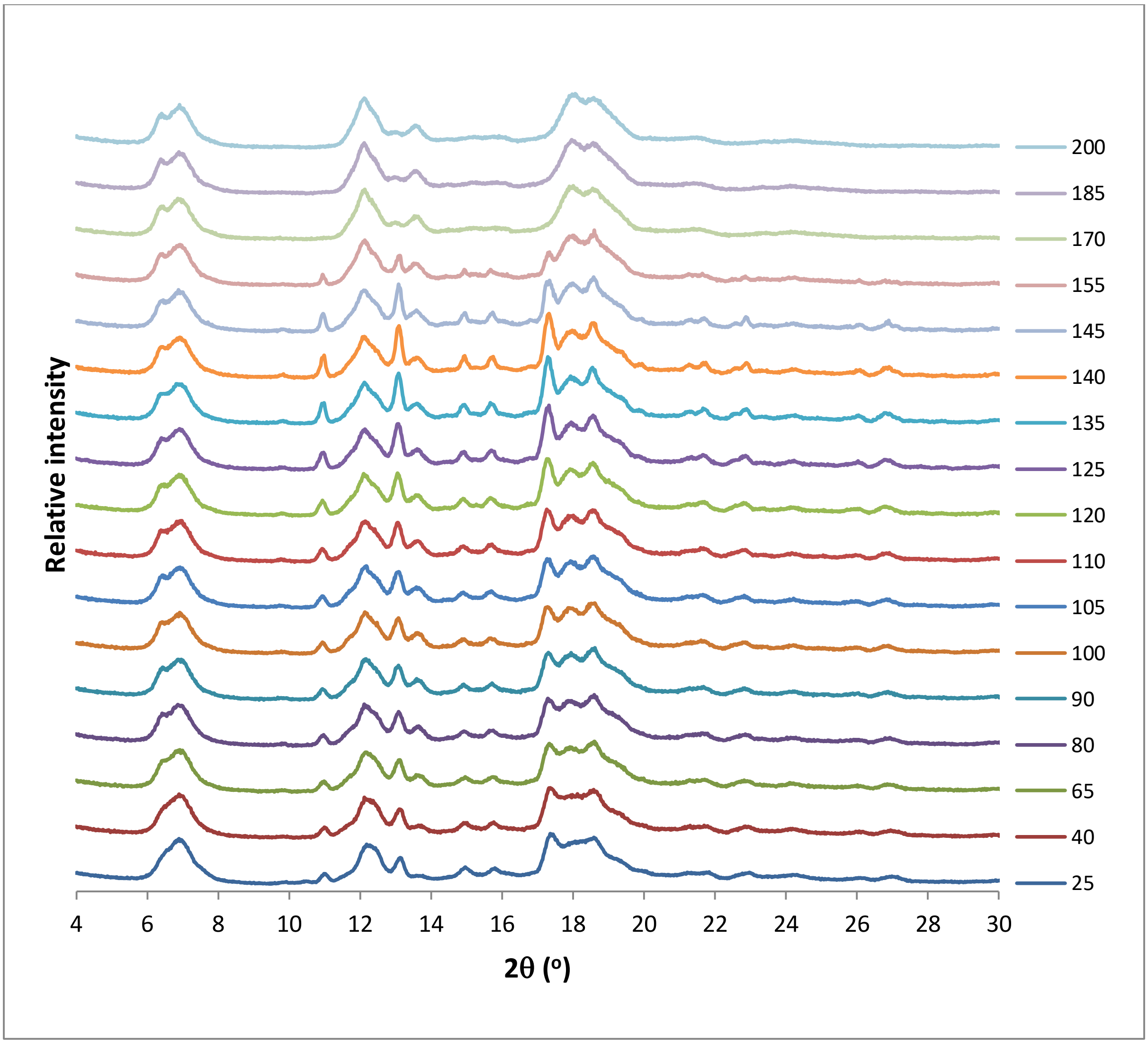

Fig.S14 The variable temperature PXRD patterns of β-CD·PRO over the temperature range from 25 °C to 200 °C. The numbers in the key represent the temperature in °C. The peak at the angular position of 7.0° is an artefact of the variable temperature PXRD stage. No significant peaks were observed at angular positions greater than 30.0°.

Section 5: Supplementary data for the TGA, dTGA and DSC analyses.

β-CD·BES: The TGA curve (Figure S15) indicated that the dehydration of the complex occurred as a multi-step process, which was confirmed by the corresponding dTGA curve (Figure S19). The dTGA curve displayed two distinctive peaks accompanied by a shoulder (on the left-hand side of the first peak), thus indicating that the water molecules were being lost at differing rates during dehydration (Figure S19). The DSC curve also reflected this multi-step dehydration process with the first two endothermic peaks (along with the shoulder in the initial broad endothermic peak) corresponding to the dehydration of β-CD·BES (Figure S15). The dehydration was shortly followed by a small exothermic event, which constituted a subtle phase transition, as confirmed by the presence of short-lived peaks in the VTPXRD patterns over this respective temperature range (Figure S13 and Table 1 [main text]).

Lastly, a sharp endothermic peak occurred, which was attributed to early decomposition. Griffith,[2] studied a β-CD inclusion complex containing naproxen and the DSC curve of this sample also featured a small, sharp endothermic peak (which was reported to occur at a high temperature of 261–273 °C) and was attributed to an initial decomposition. Since β-CD·BES and the naproxen complexes are different, it is not expected that the temperatures of the endothermic peaks necessarily corresponded; however, the type of event correlates. In addition, HSM and VTPXRD experiments (Figures 3a and S13) did not display any evidence for melting or phase transitions and therefore it is presumed that this endothermic peak in the DSC curve at an onset temperature of 213.4 $\pm$ 0.1 °C is representative of an initial decomposition (Figure S15).

β-CD·PRO: The TGA trace of β-CD·PRO also displayed a similar multi-step dehydration profile to that of β-CD·BES, the main difference being that the termination of dehydration for β-CD·PRO was more gradual, resulting in a higher dehydration termination temperature (Figure S16 and Table 1 [main text]). This possibly indicated that a few of the water molecules in β-CD·PRO have slightly stronger hydrogen bonding interactions in the complex and thus were removed only at higher temperatures. The DSC curve displayed this dehydration in the form of a broad endothermic peak with four subsequent shoulder peaks (Figure S16). The majority of the dehydration was represented by the initial broad endothermic peak, correlating with the initial step of the dehydration process in the overlaid TGA curve (Figure S16). The TGA curve (and dTGA curve, Figure S20) depicted this latter dehydration as a single step; however, the sensitivity of the DSC instrument indicated that this latter dehydration was represented by the subsequent four shoulders and thus was in fact a subtle multi-step process. This suggested that there were a few remaining water molecules that were tightly bound to the CD complex and hence only released at relatively high temperatures and at particular intervals. In addition, VTPXRD patterns indicated the presence of a phase transition, where a hardly discernible exothermic peak was observed (Figure S14). VTPXRD also confirmed that no phase transitions occurred during the dehydration of the complex.

The thermal characteristics of β-CD·BES and β-CD·PRO contained distinctive differences despite their isostructurality. Consequently, this indicated to some extent the influence that the different included guest molecules have on the thermal stability of these two inclusion complexes.

γ-CD·BES and γ-CD·PRO: The TGA profiles for γ-CD·BES and γ-CD·PRO (Figures S17 and S18) displayed very similar trends indicating single-step dehydration events for γ-CD·BES and γ-CD·PRO complexes, as confirmed by the single peak in the complementary dTGA curves (Figure S21 and S22). For both complexes the TGA curves indicated a significant decrease in the rate of mass loss towards the end of each dehydration event, as indicated by the respective shoulder peaks in the dTGA curves, indicating different binding strengths of the few remaining water molecules in each complex (Figures S21 and S22). The shoulder peak in the dTGA curve for γ-CD·BES was shallow in comparison to γ-CD·PRO (which was significantly more pronounced) suggesting subtle differences in the quantity of water molecules involved in stronger hydrogen bonding between the two complexes. In addition, the dehydration event terminated at a later temperature for γ-CD·BES in spite of having a smaller mass of water molecules, as evident in the respective TGA curves (Figures S17 and S18). This suggests that these fewer water molecules involved in the decreased rate of dehydration in γ-CD·BES had stronger hydrogen bonding interactions in comparison to γ-CD·PRO (despite their isostructurality) and thus indicates minor influences caused by the different included guest molecules. In addition, the onset of decomposition for γ-CD·PRO occurred 27.3 ^{o}C higher than for γ-CD·BES, indicating slightly improved thermal stability.

The DSC curves for γ-CD·BES and γ-CD·PRO were also similar and complemented the analysis of the TGA results (Figures S17 and S18). Both DSC curves also displayed single-step events for the dehydration, with a definitive shoulder being present for γ-CD·PRO confirming a change in the rate of dehydration, as reflected in the trend of the overlaid TGA curve and as discussed above. A shoulder was not clearly defined for γ-CD·BES; however, the asymmetry of the endothermic peak reflected the decrease in the rate of dehydration towards the end of this event for the remaining few water molecules (as also complemented by the overlaid TGA curve). In addition, no further endothermic or exothermic peaks were observed indicating thermal stability until the main decomposition event.

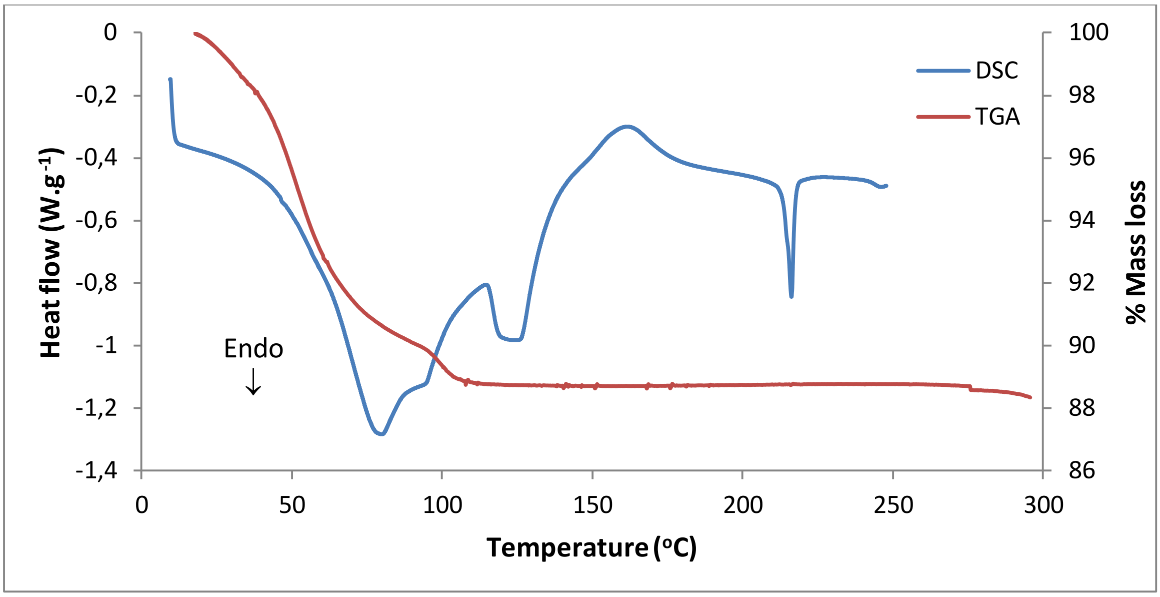

Fig.S15 A representative DSC curve (*n* = 2) of β-CD·BES, with the respective TGA curve (*n* = 3).

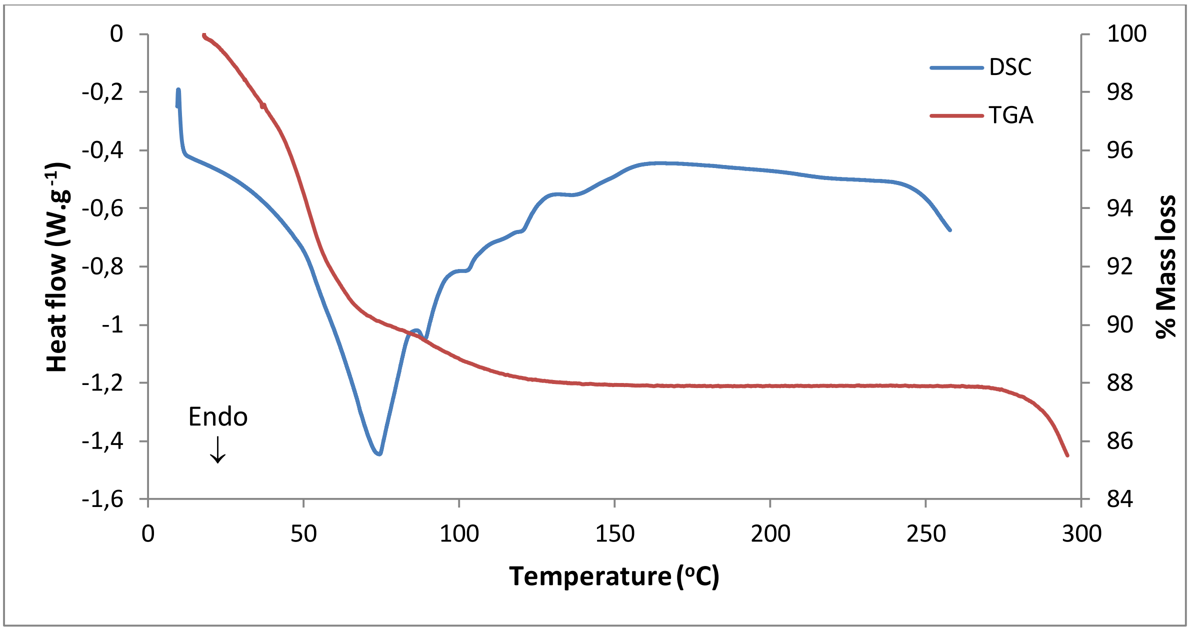

Fig.S16 A representative DSC curve (*n* = 2) of β-CD·PRO, with the respective TGA curve (*n* = 2).

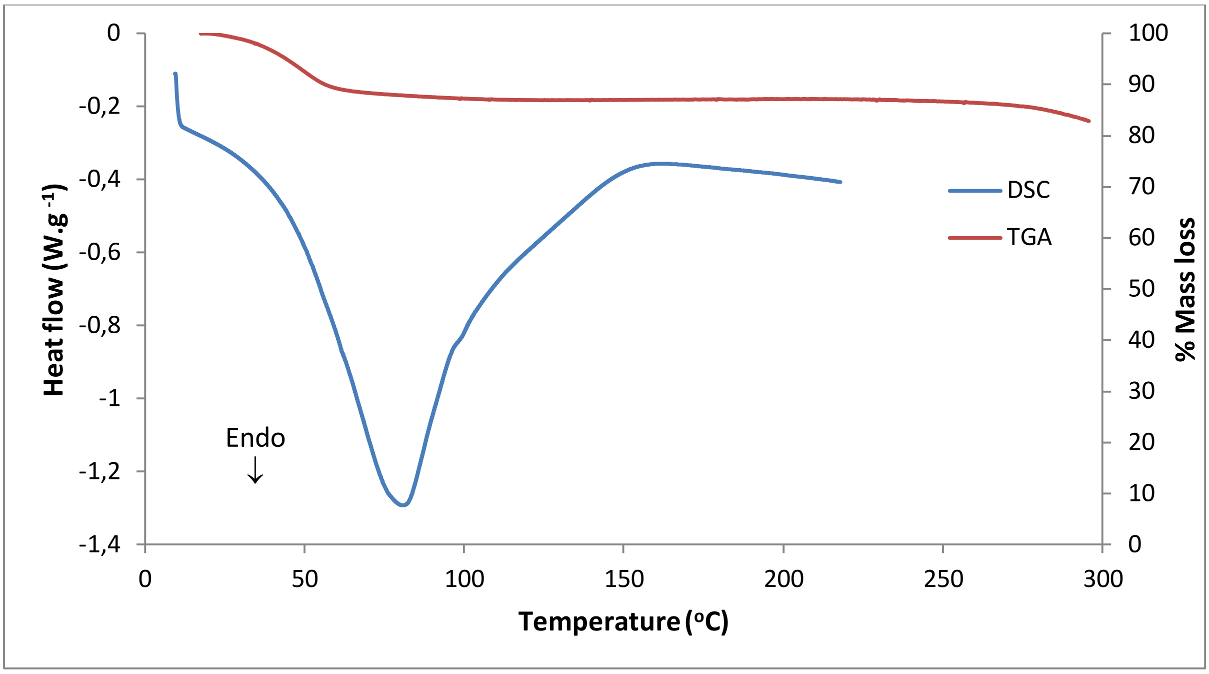

Fig.S17 A representative DSC curve of γ-CD·BES (*n* = 2), with the respective TGA curve (*n* = 2).

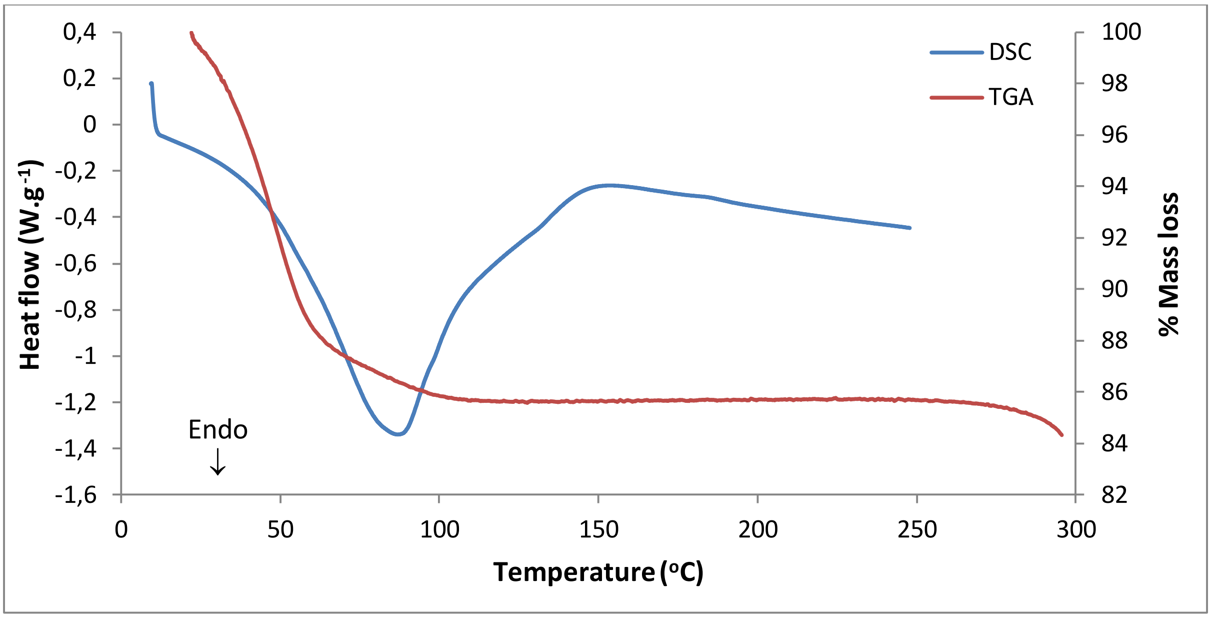

Fig.S18 A representative DSC curve of γ-CD·PRO (*n* = 3), with the respective TGA curve *n* = 3).

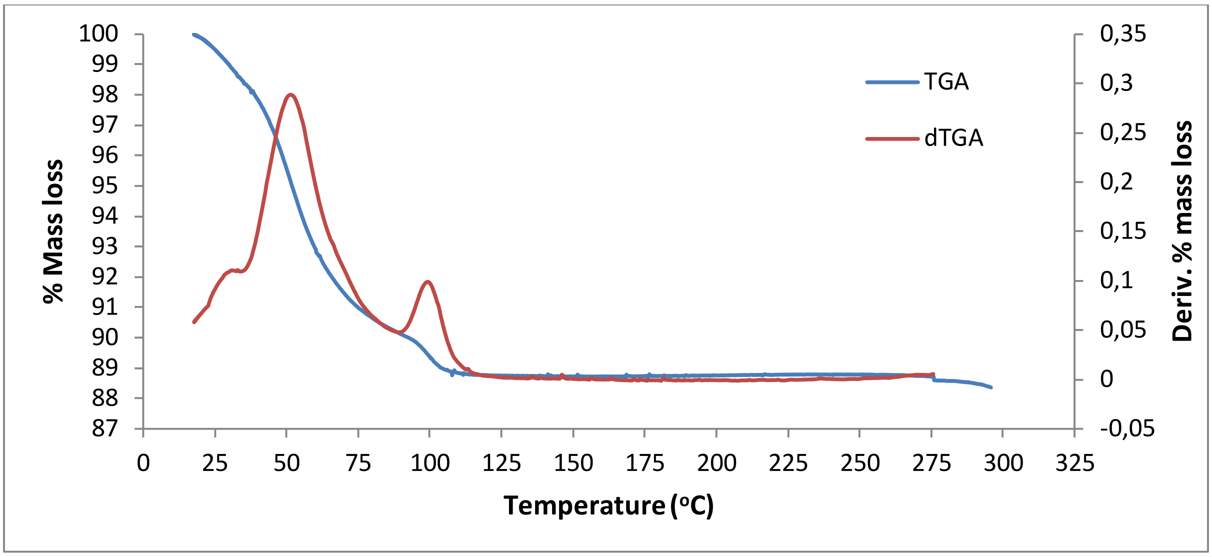

Fig.S19 Representative TGA and dTGA curves for β-CD·BES (*n* = 3).

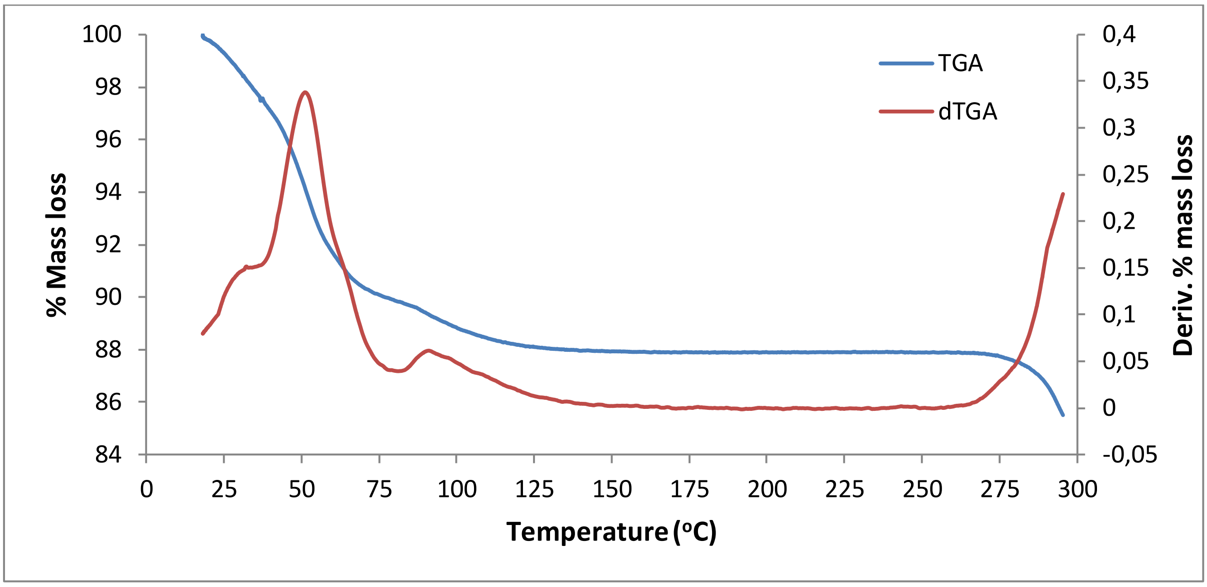

Fig.S20 Representative TGA and dTGA curves for β-CD·PRO (*n* = 2).

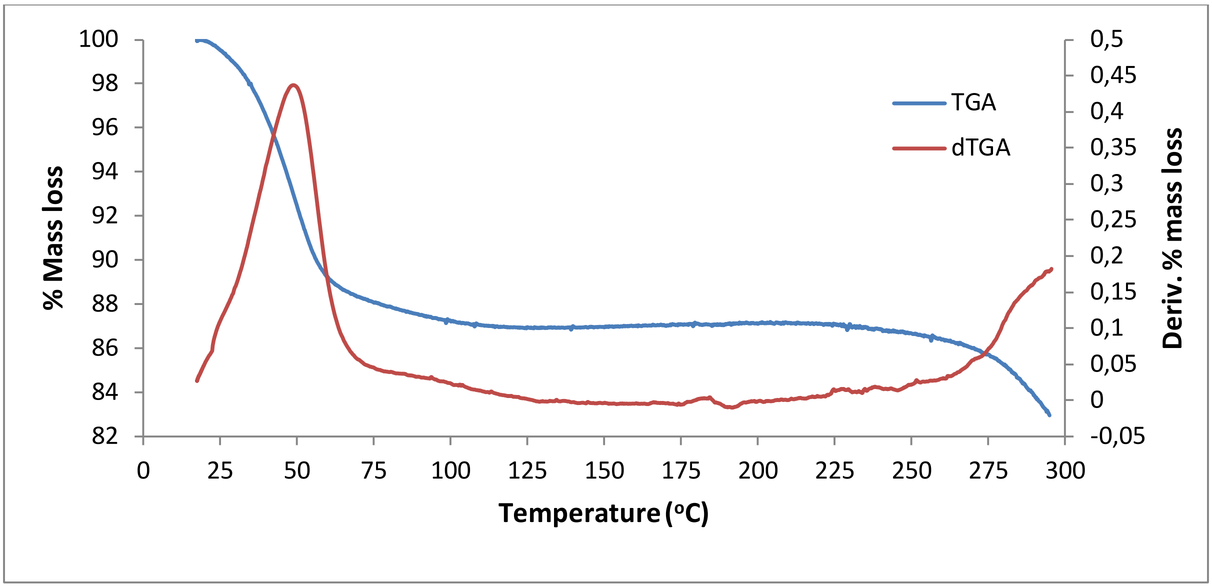

Fig.S21 Representative TGA and dTGA curves of γ-CD·BES (*n* = 2).

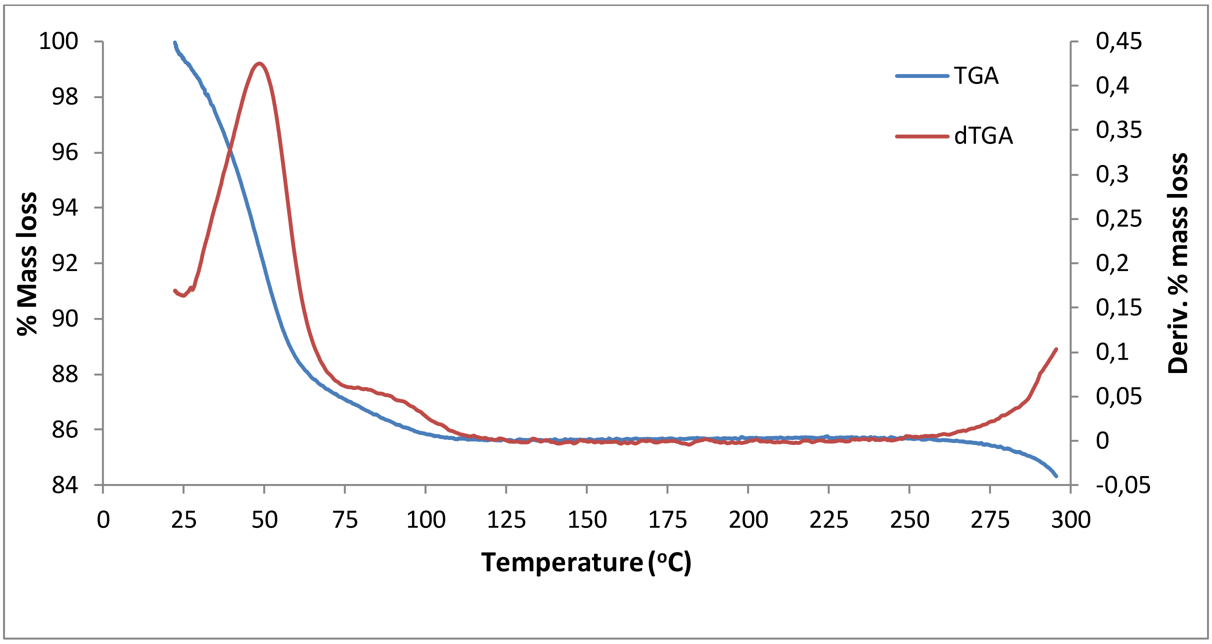

Fig,S22 Representative TGA and dTGA curves of γ-CD·PRO (*n* = 3).

Section 6: Estimating the host–guest stoichiometries of the β-CD·BES, β-CD·PRO and γ-CD·PRO complexes from electron counts derived from the Squeeze procedure.

β-CD·BES: The 399 electrons derived from the PLATON Squeeze procedure account for the guest molecule as well as the water hydrogen atoms that were not modelled in β-CD·BES. The theoretical electron count of the guest molecule (β-estradiol) and water hydrogen atom contributions per unit cell in a 2:1 host-guest complex are 296 and 70.8 electrons respectively. Therefore, in order to obtain the guest electron count contribution from the SQUEEZE procedure, the water hydrogen atom count was subtracted from the estimated total of 399 electrons per unit cell. This yielded 328.2 electrons which is in reasonable agreement with the theoretical value of 296, thus confirming the 2:1 host-guest stoichiometry determined accurately by ^{1}H NMR analysis.

β-CD·PRO: The 445 electrons derived from the PLATON Squeeze procedure account for the guest molecule as well as the water hydrogen atoms that were not modelled in β-CD·PRO. The theoretical electron count of the guest molecule (progesterone) and water hydrogen atom contributions per unit cell in a 2:1 host-guest complex are 344 and 80 electrons respectively. Therefore, in order to obtain the guest electron density contribution from the SQUEEZE procedure, the water hydrogen atom count was subtracted from the calculated total of 445 electrons per unit cell. This yielded 365 electrons, which is in excellent agreement with the theoretical value of 344, thus confirming the 2:1 host-guest stoichiometry. The latter had been determined accurately using ^{1}H NMR spectroscopy.

γ-CD·PRO: The 966 electrons derived from the PLATON Squeeze procedure account for the guest molecules as well as the water hydrogen atoms that were not modelled in γ-CD·PRO. The theoretical electron count for the guest (progesterone) molecules and water hydrogen atom contributions per unit cell in a 3:2 host-guest complex are 688 and 172.8 electrons respectively. Therefore, in order to obtain the guest electron density contribution from the SQUEEZE procedure, the water hydrogen atom count must be subtracted from the calculated total of 966 electrons per unit cell. This yields 793.2 electrons (equivalent to ~4.6 molecules of PRO per unit cell) as opposed to a theoretical value of 688 electrons (corresponding to 4.0 molecules of PRO per unit cell). Thus, the level of agreement is not reliable. However, since the stoichiometry of the complex had been determined accurately as 3:2 by ^{1}H NMR spectroscopy, reliance on the SQUEEZE estimate was not a requirement.

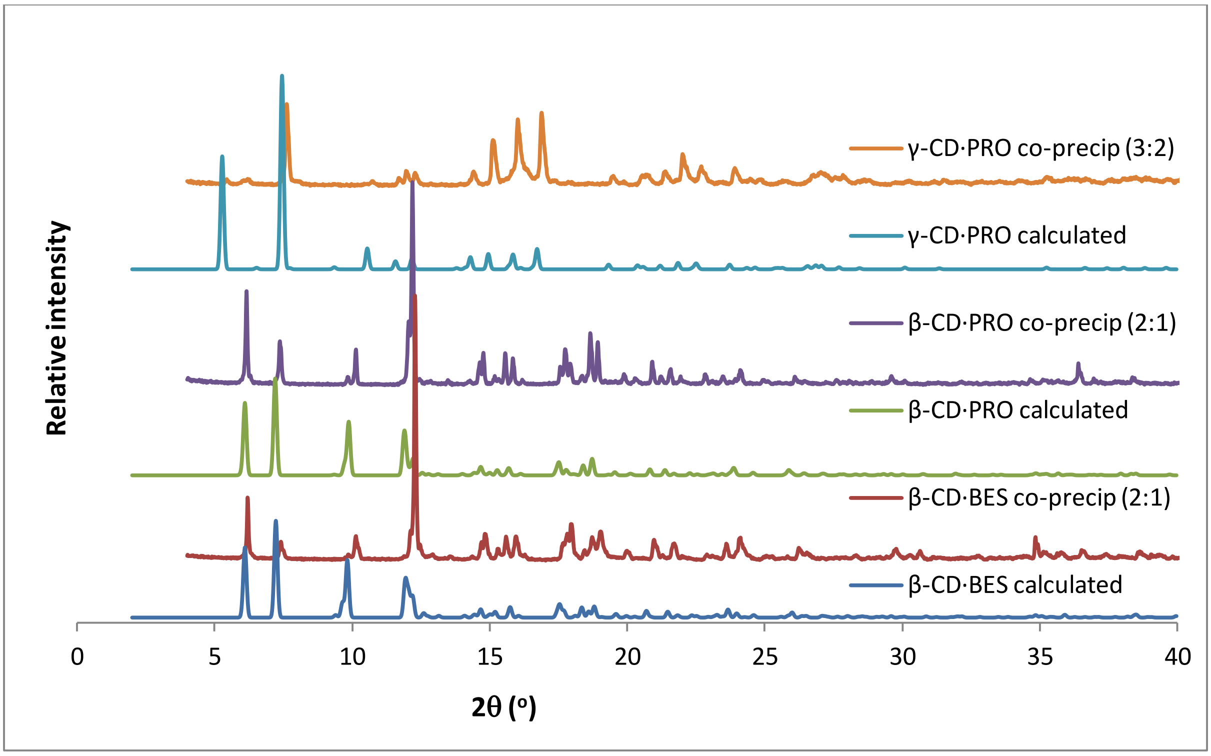

Fig.S23 The calculated PXRD patterns of β-CD·BES, β-CD·PRO and γ-CD·PRO as well as the PXRD patterns of β-CD·BES, β-CD·PRO and γ-CD·PRO prepared *via* co-precipitation.

Section 7: The geometrical parameters of the β-CD host molecules.

Table S5: The geometrical parameters of the host molecule in the complex β-CD·BES.

Residue	*l* (Å)	D (Å)	Φ (°)	d (°)	α[a] (Å)	D_3 (Å)	τ_2 (°)[b]
**A1**	5.165	4.286	125.3	2.9	0.065	2.794(7)	10.5
**A2**	4.893	4.489	131.9	-4.0	0.006	2.790(7)	4.1
**A3**	4.966	4.333	129.8	0.6	-0.079	2.783(8)	11.6
**A4**	5.211	4.311	124.4	2.1	0.045	2.809(7)	13.1
**A5**	5.003	4.439	129.8	-0.2	0.041	2.865(7)	7.7
**A6**	4.901	4.358	130.8	-2.6	-0.0461	2.808(7)	12.6
**A7**	5.078	4.355	127.9	1.4	-0.032	2.786(8)	11.9

[a] mean esd: 0.003 Å; [b] mean esd: 0.1°

Key to symbols
l : the distance between each O4 atom and the centroid of the O4-polygon;
D: the consecutive glycosidic O4···O4′ distances;
Φ: the O4···O4′···O4′′ angle;
d: the O4···O4′···O4′′···O4′′′ torsion angle;
α, the deviation of each O4 atom from the mean O4-plane;
D_3: the O2···O3′ intra-ring distance;
τ_2: the tilt angle: the angle between the mean O4-plane and the plane containing O4, C4, C1 and O4′ of a given glucose ring.

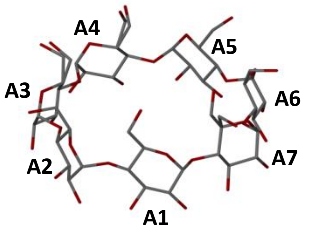

Fig.S24 The numbering scheme of the β-CD·BES glucose units.

Table S6: The geometrical parameters of the host molecule in the complex β-CD·PRO.

Residue	*l* (Å)	D (Å)	Φ (°)	d (°)	α[a] (Å)	D_3 (Å)	τ_2 (°)[b]
**A1**	4.989	4.450	130.1	-4.2	-0.005	2.781(6)	4.7
**A2**	4.994	4.350	129.1	1.8	-0.071	2.764(7)	9.8
**A3**	5.094	4.347	127.1	0.7	0.055	2.814(7)	12.5
**A4**	5.029	4.378	129.1	0.5	0.020	2.853(6)	7.7
**A5**	4.998	4.351	128.8	-3.0	-0.035	2.781(6)	10.4
**A6**	5.004	4.411	129.6	1.9	-0.028	2.787(6)	9.1
**A7**	5.116	4.285	126.2	2.3	0.064	2.757(6)	9.4

[a] mean esd: 0.003 Å; [b] mean esd: 0.1°

Key to symbols
l : the distance between each O4 atom and the centroid of the O4-polygon;
D: the consecutive glycosidic O4···O4′ distances;
Φ: the O4···O4′···O4′′ angle;
d: the O4···O4′···O4′′···O4′′′ torsion angle;
α, the deviation of each O4 atom from the mean O4-plane;
D_3: the O2···O3′ intra-ring distance;
τ_2: the tilt angle: the angle between the mean O4-plane and the plane containing O4, C4, C1 and O4′ of a given glucose ring.

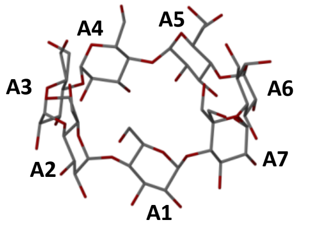

Fig.S25 The numbering scheme of the β-CD·PRO glucose units.

References

(1) Guedes, L.; Morgon, N.; Martins, M.; Pessine, F. Imiquimod/β-Cyclodextrin Inclusion Complex: Experimental and Theoretical Studies. *J. Braz. Chem. Soc.* **2020**. https://doi.org/10.21577/0103-5053.20200060.

(2) Griffith, V. J. Physicochemical Characterisation of Cyclodextrin-Drug Complexes, University of Cape Town, 1996.
